# The integrated stress response is activated in the salivary glands of Sjögren’s syndrome patients

**DOI:** 10.3389/fmed.2023.1118703

**Published:** 2023-03-23

**Authors:** Patricia Carvajal, Verónica Bahamondes, Daniela Jara, Isabel Castro, Soledad Matus, Sergio Aguilera, Claudio Molina, Sergio González, Marcela Hermoso, María-José Barrera, María-Julieta González

**Affiliations:** ^1^Programa de Biología Celular y Molecular, Instituto de Ciencias Biomédicas (ICBM), Facultad de Medicina, Universidad de Chile, Santiago, Chile; ^2^Departamento de Tecnología Médica, Facultad de Medicina, Universidad de Chile, Santiago, Chile; ^3^Edison Biotechnology Institute, Ohio University, Athens, OH, United States; ^4^Fundación Ciencia and Vida, Santiago, Chile; ^5^Facultad de Medicina y Ciencia, Universidad San Sebastián, Providencia, Santiago, Chile; ^6^Departamento de Reumatología, Clínica INDISA, Santiago, Chile; ^7^Facultad de Odontología y Ciencias de la Rehabilitación, Universidad San Sebastián, Bellavista, Santiago, Chile; ^8^Escuela de Odontología, Facultad de Medicina y Ciencias de la Salud, Universidad Mayor, Santiago, Chile; ^9^Programa de Inmunología, Instituto de Ciencias Biomédicas (ICBM), Facultad de Medicina, Universidad de Chile, Santiago, Chile

**Keywords:** Sjögren’s syndrome, acinar cells, integrated stress response, salivary glands, ATF4

## Abstract

**Introduction:**

Primary Sjögren’s syndrome (SS) is an autoimmune exocrinopathy that affects the structure and function of salivary and lachrymal glands. Labial salivary gland (LSG) acinar cells from SS patients lose cellular homeostasis and experience endoplasmic reticulum and oxidative stress. The integrated cellular stress response (ISR) is an adaptive pathway essential for restoring homeostasis against various stress-inducing factors, including pro-inflammatory cytokines, and endoplasmic reticulum and oxidative stress. ISR activation leads eIF2α phosphorylation, which transiently blocks protein synthesis while allowing the ATF4 expression, which induces a gene expression program that seeks to optimize cellular recovery. PKR, HRI, GCN2, and PERK are the four sentinel stress kinases that control eIF2α phosphorylation. Dysregulation and chronic activation of ISR signaling have pathologic consequences associated with inflammation.

**Methods:**

Here, we analyzed the activation of the ISR in LSGs of SS-patients and non-SS sicca controls, determining the mRNA, protein, and phosphorylated-protein levels of key ISR components, as well as the expression of some of ATF4 targets. Moreover, we performed a qualitative characterization of the distribution of ISR components in LSGs from both groups and evaluated if their levels correlate with clinical parameters.

**Results:**

We observed that the four ISR sensors are expressed in LSGs of both groups. However, only PKR and PERK showed increased expression and/or activation in LSGs from SS-patients. eIF2α and p-eIF2α protein levels significantly increased in SS-patients; meanwhile components of the PP1c complex responsible for eIF2α dephosphorylation decreased. ATF4 mRNA levels were decreased in LSGs from SS-patients along with hypermethylation of the ATF4 promoter. Despite low mRNA levels, SS-patients showed increased levels of ATF4 protein and ATF4-target genes involved in the antioxidant response. The acinar cells of SS-patients showed increased staining intensity for PKR, p-PKR, p-PERK, p-eIF2α, ATF4, xCT, CHOP, and NRF2. Autoantibodies, focus score, and ESSDAI were correlated with p-PERK/PERK ratio and ATF4 protein levels.

**Discussion:**

In summary, the results showed an increased ISR activation in LSGs of SS-patients. The increased protein levels of ATF4 and ATF4-target genes involved in the redox homeostasis could be part of a rescue response against the various stressful conditions to which the LSGs of SS-patients are subjected and promote cell survival.

## 1. Introduction

Primary Sjögren’s syndrome (SS) is an autoimmune, systemic, inflammatory, and chronic exocrinopathy of unknown etiology that affects the structure and function of salivary and lachrymal glands (1). Labial salivary gland (LSG) acinar cells are secretory cells subject to basal physiological stress that permanently challenges cellular homeostasis due to a high protein synthesis demand (2) and the complexity of the main secretory glycoprotein products, mucins (3). When stress exceeds the cell’s ability to handle it physiologically, it becomes pathological. Increasing evidence indicates that LSG acinar cells from SS patients lose cellular homeostasis due to altered cellular polarity, cell–cell and cell-extracellular matrix interactions, and secretory processes, among others. The secretory dysfunction include increased expression and accumulation of mucins such as MUC1 in the endoplasmic reticulum (ER) (4, 5), decreased sulfation and glycosylation of MUC5B and MUC7 (6–8), altered Ca^2+^ signaling (9), dilated ER cisternae (10), and increased pro-inflammatory cytokine expression (11) such as TNF-α and IL-6 which are associated with ER stress. ER stress strongly correlates with oxidative stress (12), which is also present in LSGs from SS-patients (13), being able to trigger a condition of pathological ER stress. Despite evident and diverse alterations and functional changes in these glands, apoptosis is not increased (14–16), suggesting the participation of adaptive mechanisms in response to cellular stress.

The integrated cellular stress response (ISR) is an adaptive pathway essential for restoring homeostasis against various stress-inducing factors (17). ISR activation leads to eIF2α (eukaryotic translation initiation factor 2 alpha) phosphorylation, which transiently blocks protein synthesis while allowing the translation of some specific mRNAs, such as the one encoding for ATF4 (activation transcription factor 4); optimizing cellular recovery (18). eIF2α phosphorylation can be induced by the activation of either of the four stress sentinel kinases: PKR (double-stranded RNA (dsRNA)-activated protein kinase), HRI (heme-regulated inhibitor), GCN2 (general control nonderepressible 2), and PERK (Protein kinase RNA-like endoplasmic reticulum kinase) (18).

**Figure 1:**
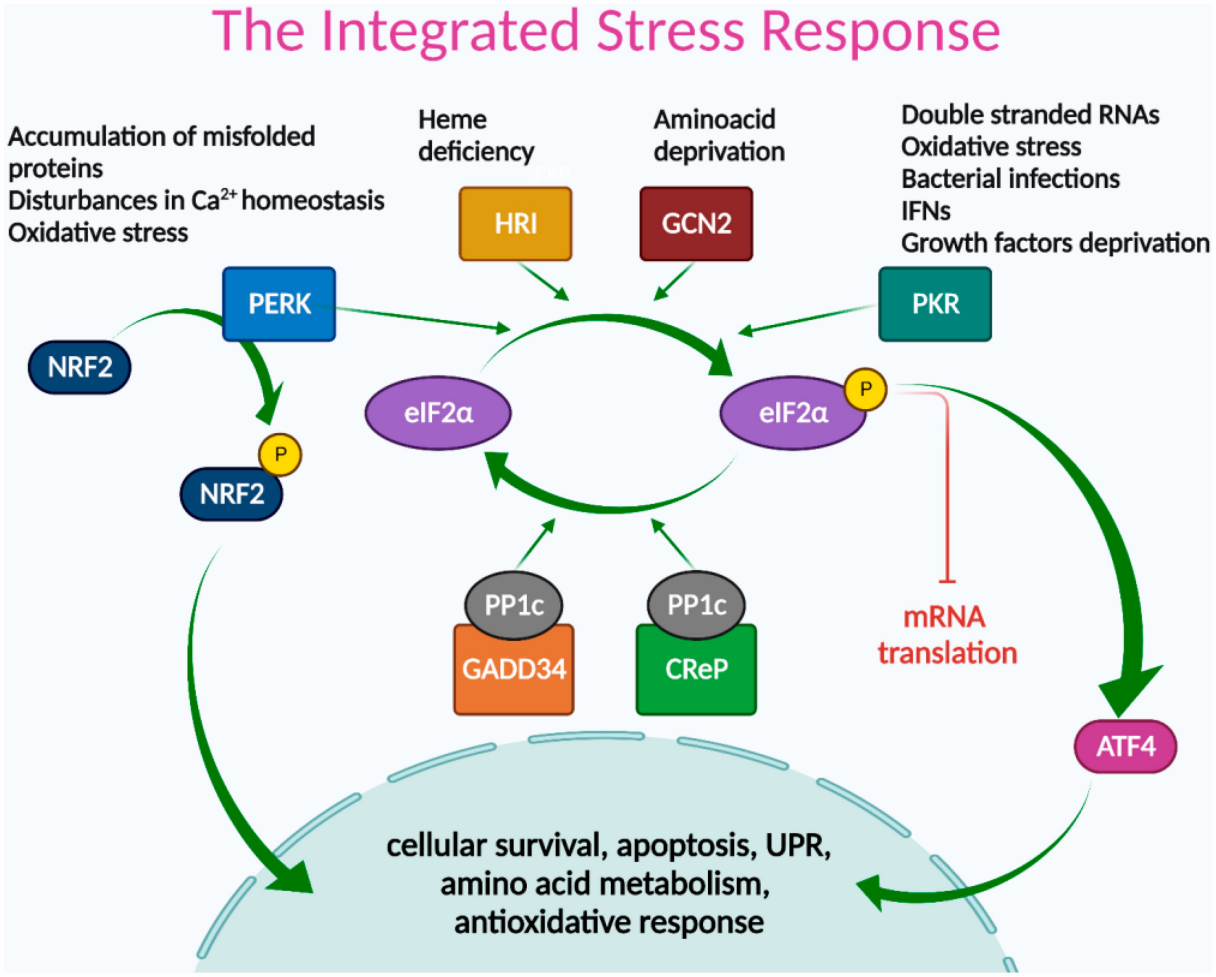
The integrated stress response signaling pathways. Different stress signals activate PERK, HRI, GCN2, or PKR kinases, which phosphorylate eIF2α, leading to global attenuation of mRNA translation and promoting the translation of ISR-specific mRNAs, such as ATF4. ATF4 transcription factor acts as a master regulator of the ISR, inducing genes involved in cellular survival, apoptosis, UPR, amino acid metabolism, and redox reactions. NRF2 is another key transcription factor of the ISR, directly activated by PERK, and mainly involved in antioxidant responses. The termination signal of the ISR is regulated by phosphatase complex PP1c/GADD34 and/or PP1c/CReP that dephosphorylate eIF2α to restore protein synthesis.

Different stressors cause sentinel kinases to dimerize, leading to autophosphorylation and activation. PKR recognizes double-stranded RNAs, and their activation inhibits the synthesis of viral and host cell proteins (18). PKR is also activated by oxidative and ER stress, pro-inflammatory cytokines such as interferons (IFNs), deprivation of growth factors, bacterial infections, caspase activity, among other stressors (19, 20). HRI is expressed primarily in erythroid cells and participates in cell differentiation during erythropoiesis. Under normal conditions, HRI binds hemin, which inhibits its protein kinase activity. Heme deficiency relieves inhibition and stimulates kinase activity, activating the ISR (18, 21). GCN2 is activated by binding to deacylated transfer RNAs (tRNAs) through its histidyl-tRNA synthetase-related domain, which accumulates in response to amino acid deprivation (22). PERK is located in the ER membrane and is activated by ER stress, caused by the accumulation of misfolded proteins, disturbances in calcium homeostasis or redox state, among others (23). PERK is also one of the three sensors of the unfolded protein response (UPR) that recovers and preserves cellular homeostasis under ER stress conditions (24). LSGs of SS-patients showed altered activation of two UPR sensors: low IRE1α/XBP-1 (25) and increased ATF6α signaling pathway activity (16). PERK expression and activation in LSGs of SS-patients have not yet been studied. During ER stress, PERK phosphorylates eIF2α and can also phosphorylate a key ISR transcription factor, NRF2 (Nuclear erythroid 2-Related Factor 2). In basal conditions, NRF2 is sequestered by cytoplasmic KEAP1 (Kelch Like ECH Associated Protein 1) and targeted to proteasomal degradation. However, upon oxidative stress, the interaction between NRF2 and KEAP1 is disrupted, and NRF2 translocates to the nucleus regulating genes with antioxidant response elements (ARE) in their promoters, promoting cell survival (26, 27). Since the different cells of an organism are exposed to different stressors, it is expected that only some of these kinases are simultaneously active in the same cell (Figure 1).

ATF4 acts as a master regulator of the ISR, inducing a gene expression program dependent on the cellular context and stress intensity, mediated by its ability to dimerize with other transcription factor partners and its regulation at the transcriptional, translational, and post-translational levels (18). In general terms, ATF4 regulates the expression of genes involved in cellular survival, apoptosis, UPR, amino acid metabolism, and redox reactions (Figure 1) (28).

The ending signal of the ISR is the dephosphorylation of eIF2α to restore protein synthesis. This is mediated by the Protein Phosphatase 1 (PP1) complex, which recruits the catalytic PP1c subunit and one of its two regulatory subunits: CREP or GADD34. CREP is constitutively expressed, while GADD34 is expressed in stressed cells (18).

Dysregulation and chronic activation of ISR signaling have pathologic consequences associated with inflammation (29). For example, retinal cells from a murine model of diabetic retinopathy developed ER stress and cytokine production dependent on ATF4 (30). PKR plays a pro-inflammatory role, exerting its regulatory effects by modulating diverse signaling pathways (31–34). For example, PKR-deficient fibroblast showed that the PKR sensor participates in the NF-κB signaling pathway independently of its kinase function after interacting with the IKK (31), inducing the expression of pro-inflammatory cytokines. In macrophages, ISR inhibitors suppress inflammation and inflammasome activation induced by oxidative stress, toll-like receptor (TLR) agonists (33), and hyperlipidemia (34).

Here, we analyze the activation of the ISR in LSGs of SS-patients, determining the mRNA, protein, and phosphorylation levels of the stress sensor kinases, eIF2α, ATF4, NRF2, and the protein phosphatase complex components involved in eIF2α dephosphorylation. Since ATF4 mediates diverse responses to cellular stress, we also evaluated the expression of some of its targets, such as components of the system Xc-involved in glutathione (GSH) synthesis, the anti-apoptotic molecule survivin, and the pro-apoptotic molecule CHOP (transcription factor C/EBP homologous protein). We also performed a qualitative characterization of ISR component distribution in LSGs from controls and SS-patients and evaluated if the levels of ISR components correlate with clinical parameters.

## 2. Materials and methods

### 2.1. Patients with primary SS and controls

SS-patients (*n* = 41) were diagnosed according to the 2016 American College of Rheumatology/European League against Rheumatism Classification Criteria (ACR/EULAR) (35). The control group (*n* = 34) consisted of non-SS sicca subjects selected from individuals who had consulted their doctor because of oral and/or ocular dryness symptoms, but who did not fulfill the primary SS classification criteria. They did not have systemic diseases, and their lip biopsy analysis was established as normal or mild diffuse chronic sialadenitis. Moreover, they were negative for rheumatoid factor and anti-Ro and anti-La antibodies. Supplementary Table S1 summarizes the demographic, serological, and histological characteristics of SS-patients and controls. Written consent was obtained according to the Declaration of Helsinki. The Ethical Committee of the Faculty of Medicine, University of Chile approved this study (N° 001-2021).

### 2.2. Biopsies

LSGs were obtained for diagnostic purposes from SS-patients and controls as previously described (36). Once the samples were obtained, they were split into two portions; one was snap-frozen in liquid nitrogen and stored at −80°C until processed. The other was fixed for immunofluorescence studies.

### 2.3. RNA extraction, reverse transcription, and real-time PCR

Yields and purity of total RNA were evaluated as previously described (37). Relative mRNA levels were determined by semi-quantitative real-time PCR (16), using primers designed with AmplifX 1.4 software (Supplementary Table S2). Target gene transcript levels were normalized to h18S using the efficiency-calibrated model (38).

### 2.4. Methylation sensitive high-resolution melting analysis

Genomic DNA was extracted from LSG from SS-patients (*n* = 10) and control subjects (*n* = 5) using the All Prep kit (Qiagen, United States) and treated with MethylCode™ Bisulfite Conversion kit (Invitrogen, Carlsbad, CA, United Sates). MS-HRM primers for the ATF4 gene promoter analysis were designed using Methyl Primer Express Software v1.0 (Applied Biosystems) (Supplementary Table S2). Next, PCR amplification of bisulfite-modified templates was performed as previously described in detail (25).

### 2.5. Immunofluorescence

Immunofluorescence analysis was employed to detect the subcellular distribution of PKR, p-PKR, PERK, p-PERK, eIF2α, p-eIF2α, ATF4, xCT, CHOP, and NRF2. LSGs were fixed in 1% (w/v) p-formaldehyde and embedded in paraffin. The obtained sections were subjected to antigen recovery by incubation with a 0.01 M citrate solution, pH 6.0, and incubated overnight at 4°C with the previously validated primary antibodies (Supplementary Table S3). Subsequently, samples were incubated with Alexa Fluor 488-conjugated secondary antibody and Hoechst 33342 for nuclear staining. Sections were mounted in Mowiol. Immunofluorescence was visualized with an Olympus FluoView FV10i confocal laser scanning microscope (Olympus, United States) or C2 confocal laser scanning microscope (Nikon, Tokyo, Japan). High-resolution digital images were captured and stored in TIFF format. As a negative control, IgG was employed (DakoCytomation, Inc. CA, United States). Three observers qualitatively evaluated the immunofluorescence images to determine the distribution of ISR components in different glandular components such as acini, ducts, or inflammatory cells. The observers were previously trained and calibrated among themselves. Cohen’s kappa coefficients inter-rater and intra-rater reliability were greater than 0.8.

### 2.6. Protein extraction and western blotting

LSG samples were homogenized as previously described (37). Proteins were quantified by the bicinchoninic acid method and separated by SDS-PAGE in 8% gels under reducing and denaturing conditions. The separated proteins were transferred to nitrocellulose membranes and then blocked in 5% (w/v) skimmed milk (protease-free) prepared in TBS-T (10 mM Tris HCl (pH 7.5), 150 mM NaCl, 0.1% (v/v) Tween-20). Subsequently, blots were separately incubated with primary antibodies against PKR, p-PKR, HRI, GCN2, PERK, p-PERK, eIF2α, p-eIF2α, PP1c, GADD34, CREP, ATF4, xCT, CHOP, survivin, NRF2, p-NRF2, KEAP1, and β-actin according to data from Supplementary Table S3. After washing in TBS-T, blots were incubated for 1 h at room temperature with goat anti-mouse or anti-rabbit peroxidase-conjugated secondary antibodies, pre-absorbed on a solid-phase carrier with immobilized human serum proteins to ensure minimal non-specific signal (Pierce). Protein bands were visualized by enhanced chemiluminescence (Pierce) and quantified by densitometry. Protein levels were normalized to the values obtained for β-actin.

### 2.7. Statistical analysis

Mean values in control and SS-patient groups were compared using the Mann–Whitney test. Spearman’s rank correlation analysis was also performed. *p* values lower than 0.05 were considered significant.

## 3. Results

### 3.1. Expression and activation of ISR kinases in LSGs from SS-patients

**Figure 2:**
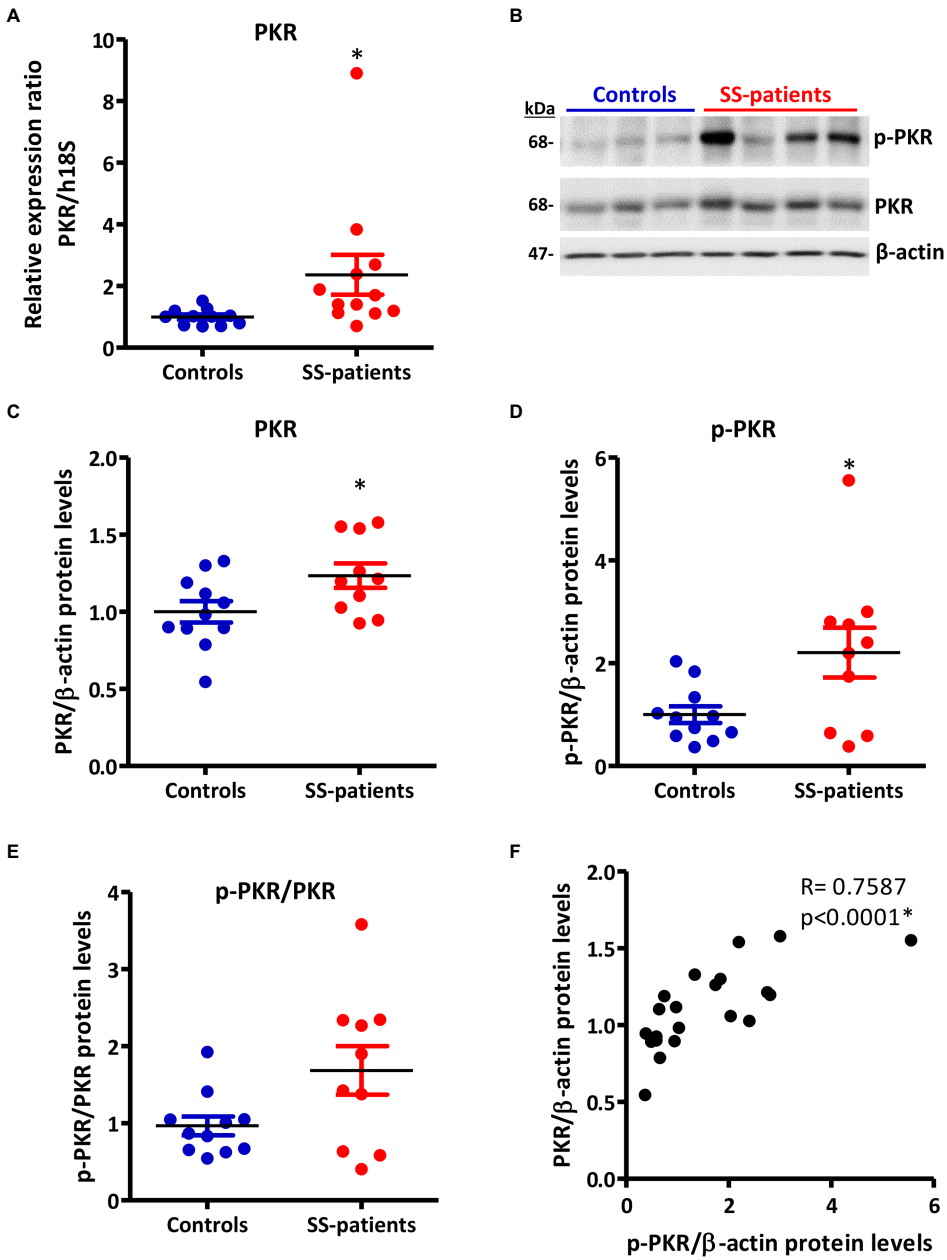
Expression and activation of PKR in LSGs from controls and SS-patients. **(A)** Dot plot showing PKR transcript levels relative to h18S in LSGs from controls (C) and SS-patients (P) (*n* = 11C, 12P). **(B)** Representative images of PKR and p-PKR immunoblots from controls and SS-patients using β-actin as a loading control. **(C)** Dot plot showing densitometric analysis of PKR (*n* = 11C, 10P). **(D)** Dot plot showing densitometric analysis of p-PKR (*n* = 11C, 10P). **(E)** Dot plot showing the p-PKR/PKR ratio (*n* = 11C, 10P). **(F)** Spearman’s correlation between p-PKR and PKR protein levels. These experiments were repeated at least three times (*). *p* values lower than 0.05 were considered significant.

**Figure 3:**
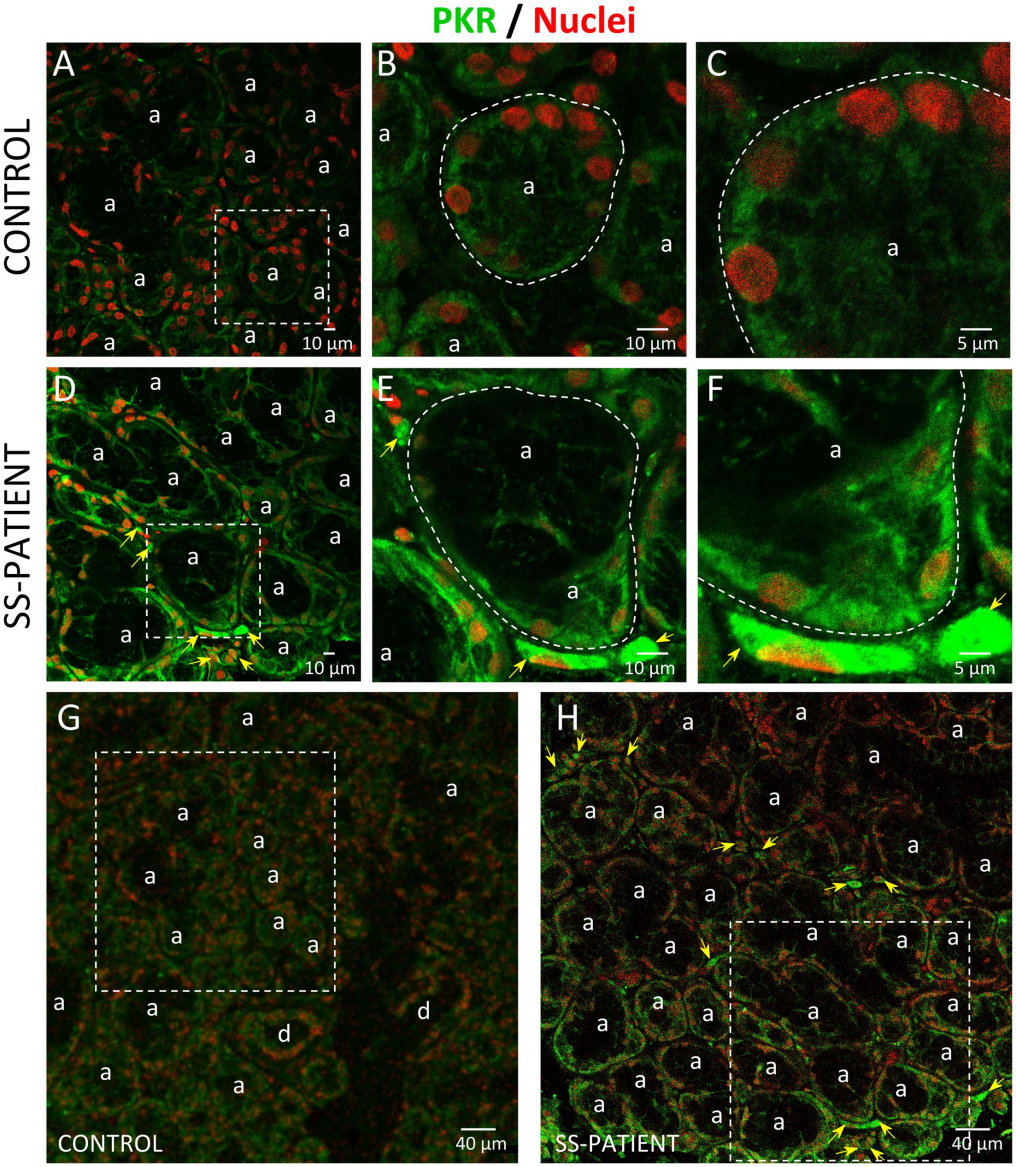
Localization of PKR in LSGs from controls and SS-patients. **(A–C,G)** PKR (green) staining was mainly observed in the basolateral cytoplasm of epithelial cells in LSG from control subjects. **(D–F,H)** Stronger PKR (green) staining was observed in the cytoplasm of epithelial and plasma cells (yellow arrows) in LSGs from SS-patients. **(A,D)** Higher magnifications of regions bounded by broken lines in G and H, respectively. **(B,C,E,F)** Higher magnifications of regions bounded by broken lines in A and B, respectively. Nuclei (red) were counterstained with Hoechst-33342. a: acini. Bars A, B, D and E: 10 μm; C and F: 5 μm; G and H: 40 μm.

**Figure 4:**
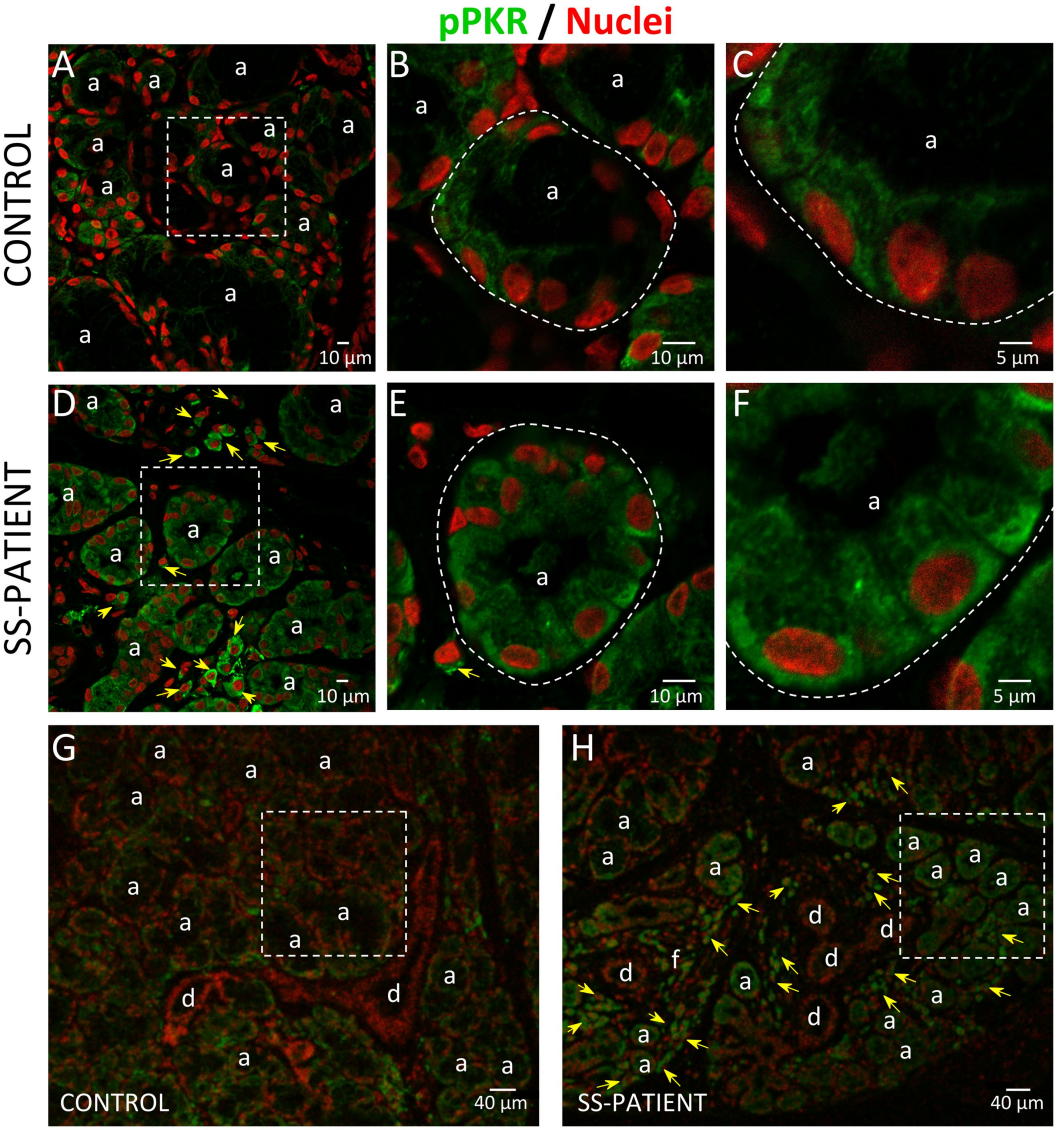
Localization of p-PKR in LSGs from controls and SS-patients. **(A–C,G)** p-PKR (green) staining was mainly observed in the cytoplasm of epithelial cells in LSGs from control subjects. **(D–F,H)** Stronger p-PKR (green) staining was observed in the cytoplasm of epithelial cells and plasma cells (yellow arrows) in LSGs from SS-patients. **(A,D)** Higher magnifications of regions surrounded by broken lines in G and H, respectively. **(B,C,E,F)** Higher magnifications of regions surrounded by broken lines in A and B, respectively. Nuclei (red) were counterstained with Hoechst-33342. a: acini; d: duct; f: focus of inflammatory cells. Bars A, B, D, and E 10: μm; C and F: 5 μm; G and H: 40 μm.

Activation of ISR leads to eIF2α phosphorylation, which transiently repress protein synthesis and is controlled by four sentinel stress kinases: PKR, HRI, GCN2, and/or PERK. PKR can be activated by dsRNA, oxidative and ER stress, pro-inflammatory cytokines such as IFNs, deprivation of growth factors, bacterial infections, caspase activity, and their activation inhibits protein synthesis including viral proteins (39). In LSGs from SS-patients, we observed a significant increase in PKR mRNA levels (*p* = 0.0031, Figure 2A), which positively correlated with Ro autoantibodies and with the degree of glandular inflammation (focus score) (Supplementary Table S4). PKR protein levels also significantly increased in SS-patients (*p* = 0.0264) (Figures 2B,C). Phosphorylation of these kinases is the parameter that defines their activation; therefore, we measured phosphorylated protein levels by Western blot. Phosphorylated PKR (p-PKR) protein levels significantly increased (*p* = 0.0363) in LSGs from SS-patients (Figures 2B,D) without changes in the p-PKR/PKR ratio (Figure 2E). Additionally, p-PKR protein levels strongly correlated with PKR protein levels (Figure 2F) and PERK protein levels (Supplementary Table S4). Real-time PCR and Western blot were performed using total glandular extracts; however, they do not reflect the spatial distribution of the studied molecules. To qualitatively characterize the distribution of ISR components in different glandular structures or cell types, immunofluorescence analyses were carried out in sections of LSGs from SS-patients and controls. The detection of PKR and p-PKR showed a cytoplasmic localization in acinar cells with stronger immunofluorescent staining in LSGs of SS-patients (Figures 3, 4) PKR and p-PKR were also strongly detected in plasma cells, while no staining was observed in duct cells (Figures 3, 4). These results suggest that PKR activation is increased in acinar cells of LSGs from SS-patients.

HRI is activated by iron deprivation in erythroid cells (21) and by oxidative stress (40), heat shock, osmotic stress, and nitric oxide, among others (41). There were no differences in HRI transcript (*p* = 0.20) and protein levels (*p* = 0.22) between controls and SS-patients (Supplementary Figures S1A–C). Additionally, phosphorylated HRI could not be detected since there are no commercial antibodies against p-HRI.

GCN2 is activated in response to amino acid deprivation (22). There were no differences in GCN2 transcripts levels (*p* = 0.49) between controls and SS-patients (Supplementary Figure S2A), and GCN2 and p-GCN2 protein levels were unchanged in SS-patients compared to controls (*p* = 0.37 and *p* = 0.24, respectively) (Supplementary Figures S2B–D). The p-GCN2/GCN2 ratio was similar between SS-patients and controls (Supplementary Figure S2E), and there was a strong correlation between p-GCN2 and GCN2 protein levels (Supplementary Figure S2F).

**Figure 5:**
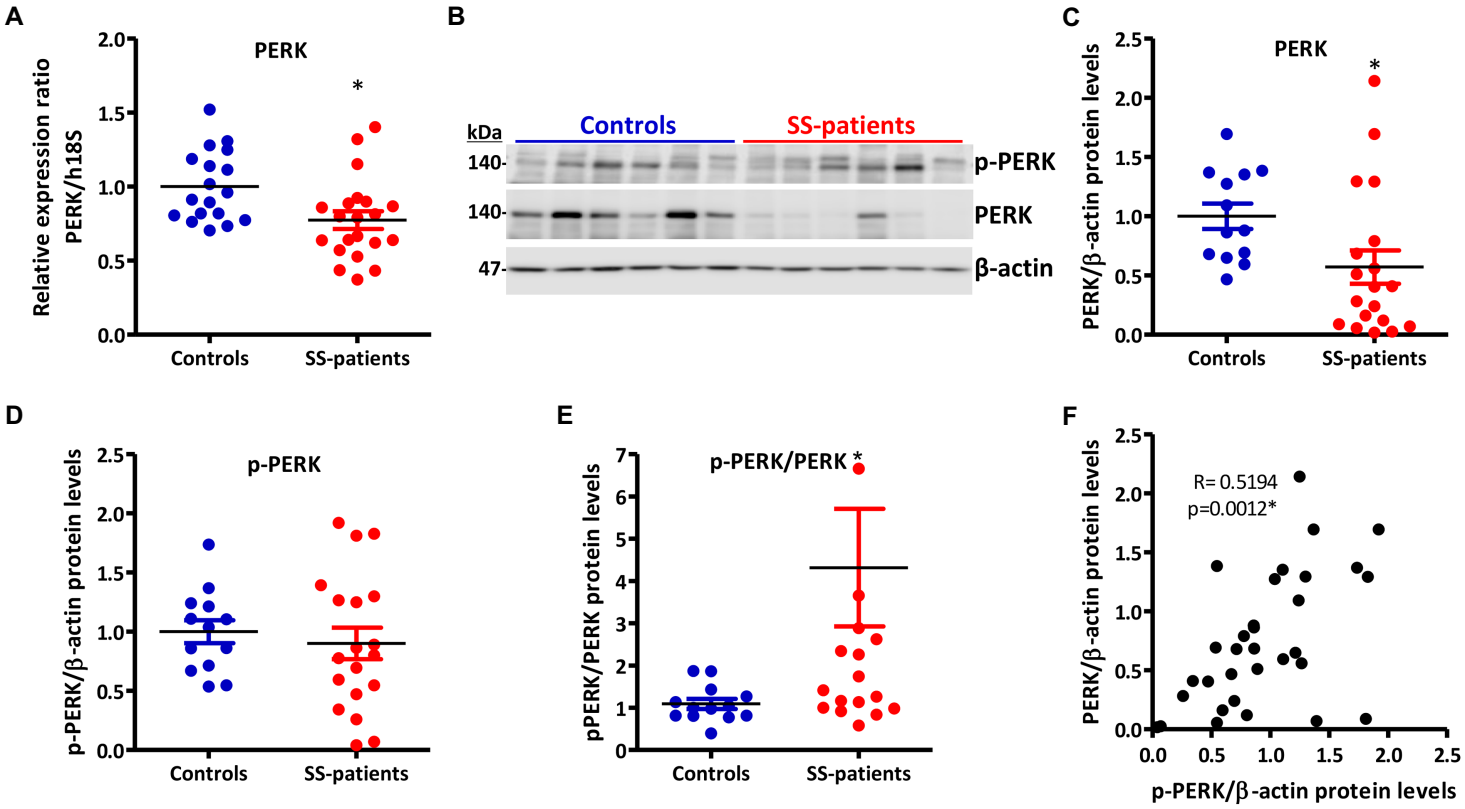
Expression and activation of PERK in LSGs from control and SS-patients. **(A)** Dot plot showing PERK transcript levels relative to h18S from control (C) and SS-patients (P) (*n* = 18C, 21P). **(B)** Representative images of p-PERK and PERK immunoblots from control and SS-patients using β-actin as a loading control. **(C)** Dot plot showing densitometric analysis of PERK (*n* = 13C, 19P). **(D)** Dot plot showing densitometric analysis of p-PERK (*n* = 13C, 19P). **(E)** Dot plot showing the p-PERK/PERK ratio (*n* = 13C, 19P). **(F)** Spearman’s correlation between p-PERK and PERK protein levels. These experiments were repeated at least three times (*). *p* values lower than 0.05 were considered significant.

**Figure 6:**
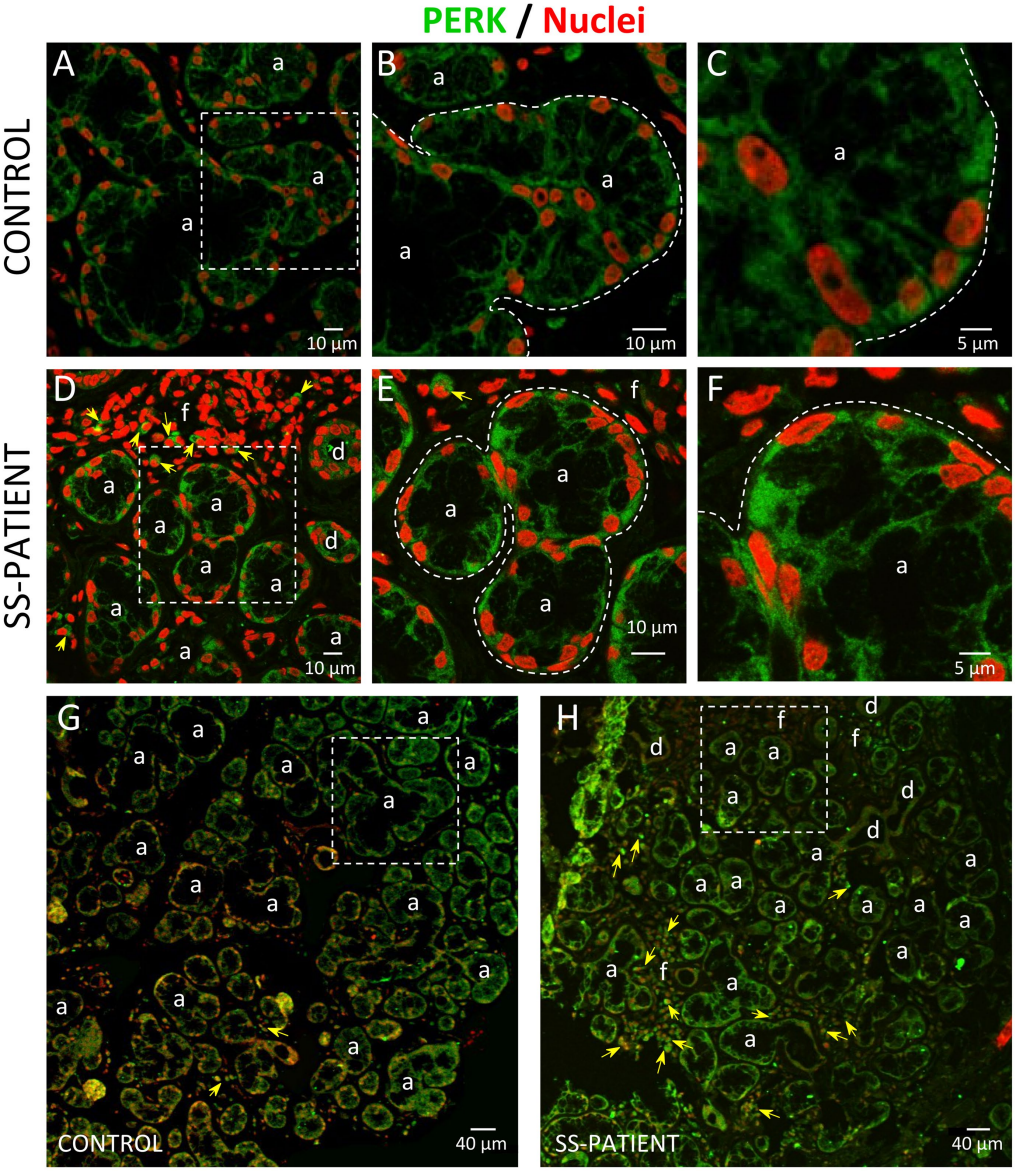
Localization of PERK in LSGs from control and SS-patients. **(A–C,G)** PERK (green) staining was mainly observed in the basolateral cytoplasm of epithelial cells in LSG from control subjects. **(D–F,H)** PERK (green) staining was also observed in the basolateral cytoplasm of epithelial cells and few plasma cells (yellow arrows) in LSGs from SS-patients. **(A,D)** Higher magnifications of regions surrounded by broken lines in G and H, respectively. **(B,C,E,F)** Higher magnifications of regions surrounded by broken lines in A and B, respectively. Nuclei (red) were counterstained with Hoechst-33342. a: acini; d: duct; f: focus of inflammatory cells. Bars A, B, D and E: 10 μm; C and F: 5 μm; G and H: 40 μm.

**Figure 7:**
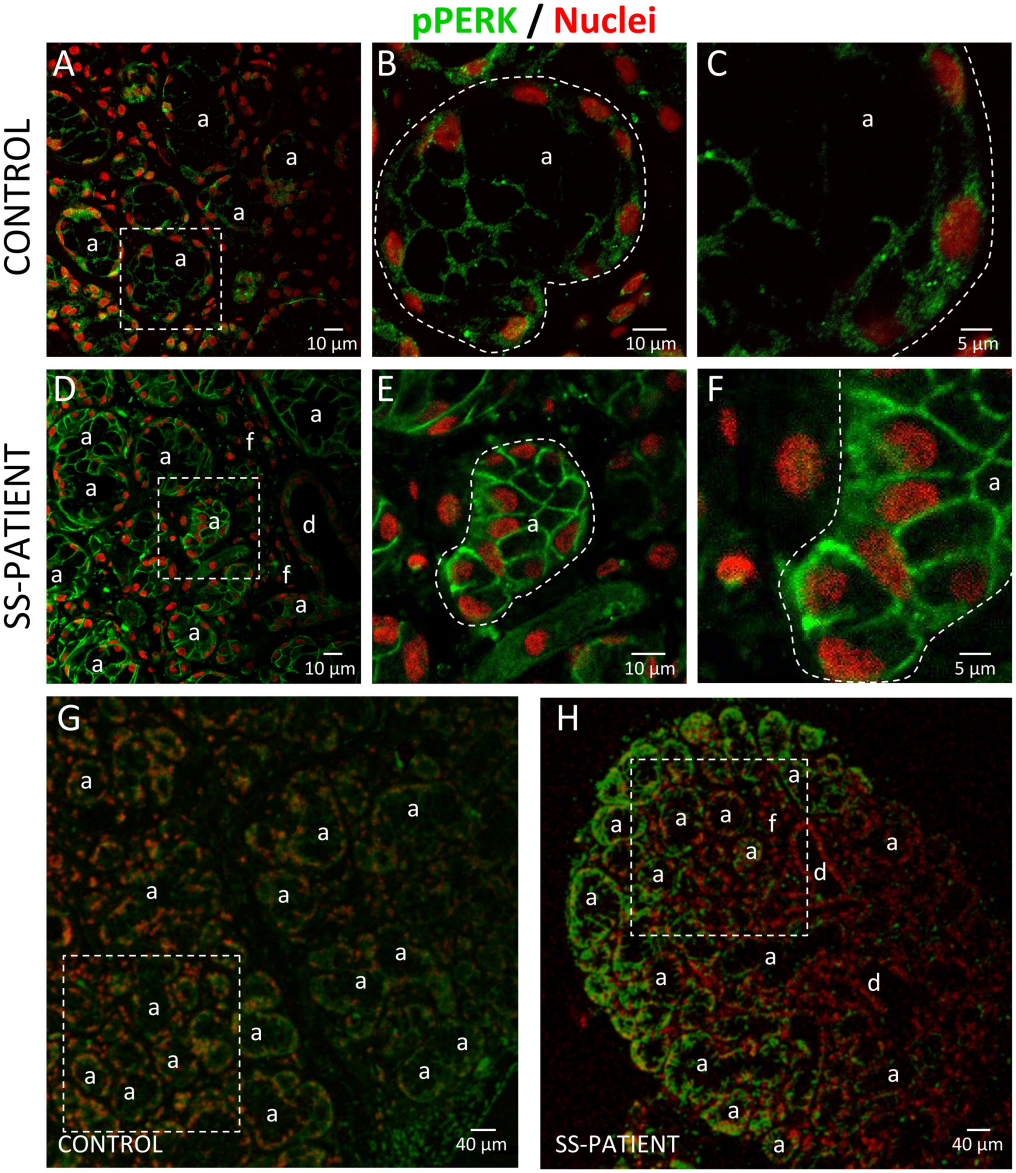
Localization of p-PERK in LSGs from control and SS-patients. **(A–C,G)** p-PERK (green) staining was mainly observed in the basolateral cytoplasm of epithelial cells in LSGs from control subjects. **(D–F,H)** Stronger p-PERK (green) staining was observed in the basolateral cytoplasm and plasma membrane of epithelial cells in LSGs from SS-patients. **(A,D)** higher magnifications of regions surrounded by broken lines in G and H, respectively. **(B,C,E,F)** Higher magnifications of regions surrounded by broken lines in A and B, respectively. Nuclei (red) were counterstained with Hoechst-33342. a: acini; d: duct; f: focus of inflammatory cells. Bars A, B, D and E: 10 μm; C and F: 5 μm; G and H: 40 μm.

PERK is activated by the accumulation of misfolded/unfolded proteins in ER (42). In LSGs from SS-patients, we observed a significant decrease in PERK mRNA levels (*p* = 0.011, Figure 5A) and PERK protein levels (*p* = 0.0045, Figures 5B,C). PERK is phosphorylated (p-PERK) in both LSGs of SS-patients and controls, and its phosphorylation levels were similar (*p* = 0.323) in both groups (Figures 5B,D), despite the low total PERK protein levels in SS-patients. This could explain the significant increase in the p-PERK/PERK ratio (*p* = 0.0014) in SS-patients (Figure 5E). p-PERK protein levels positively correlated with PERK protein levels (Figure 5F). The p-PERK/PERK ratio positively correlated with the presence of autoantibodies (Ro, La, ANA, RF), the degree of glandular inflammation (focus score), ESSDAI, and inversely correlated with the unstimulated whole salivary flow (UWSF) (Supplementary Table S4). PERK (Figure 6) and p-PERK (Figure 7) staining was mainly observed in the basal region of acinar cells, where the ER localizes. Some plasma cells show immunostaining for PERK, but most inflammatory cells did not (Figure 6D). Therefore, the decreased PERK protein levels observed by Western blot could be due to a dilution effect caused by the presence of inflammatory cells in LSGs from SS-patients. p-PERK levels were unchanged between groups *via* Western blot, and the p-PERK/PERK ratio was increased in LSGs from SS-patients. This is because PERK is mainly phosphorylated in LSGs from SS-patients as shown by immunofluorescence, suggesting activation of this pathway.

Together these results demonstrate that all four ISR sensors are expressed in LSGs, but only PKR and PERK activation is increased in LSGs from SS-patients, supporting the presence of a pathological cellular stress condition.

### 3.2. Expression and activation of eIF2α in LSGs from SS-patients

**Figure 8:**
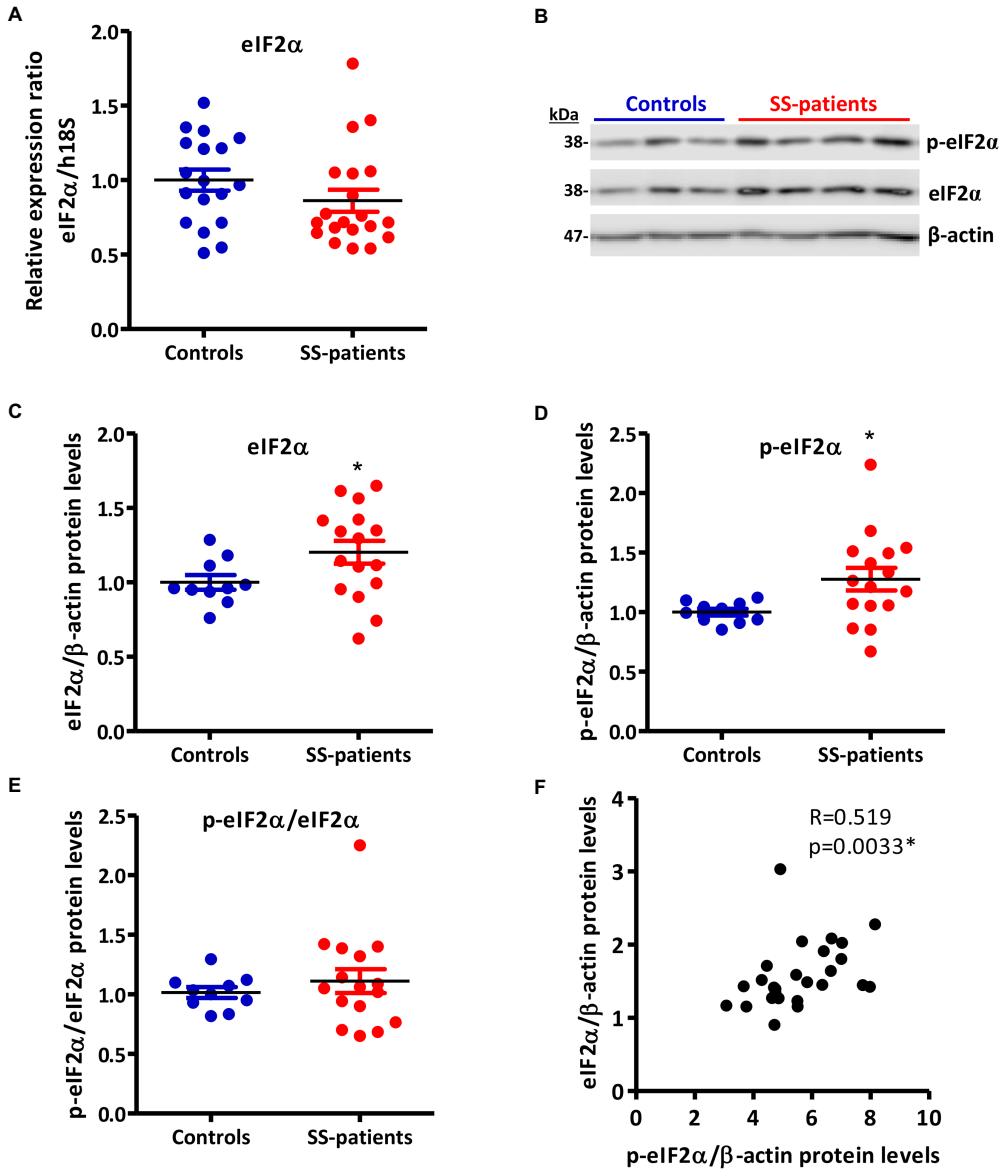
Expression of eIF2α and p-eIF2α in LSGs from control and SS-patients. **(A)** Dot plot showing eIF2α transcript levels relative to h18S from controls (C) and SS-patients (P) (*n* = 18C, 20P). **(B)** Representative images of p-eIF2α and eIF2α immunoblots from control and SS-patients using β-actin as a loading control. **(C)** Dot plot showing densitometric analysis of eIF2α (*n* = 10C, 16P). **(D)** Dot plot showing densitometric analysis of p-eIF2α (*n* = 10C, 16P). **(E)** Dot plot showing the p-eIF2α/eIF2α ratio (*n* = 10C, 16P). **(F)** Spearman’s correlation between p-eIF2α and eIF2α protein levels. These experiments were repeated at least three times (*). *p* values lower than 0.05 were considered significant.

**Figure 9:**
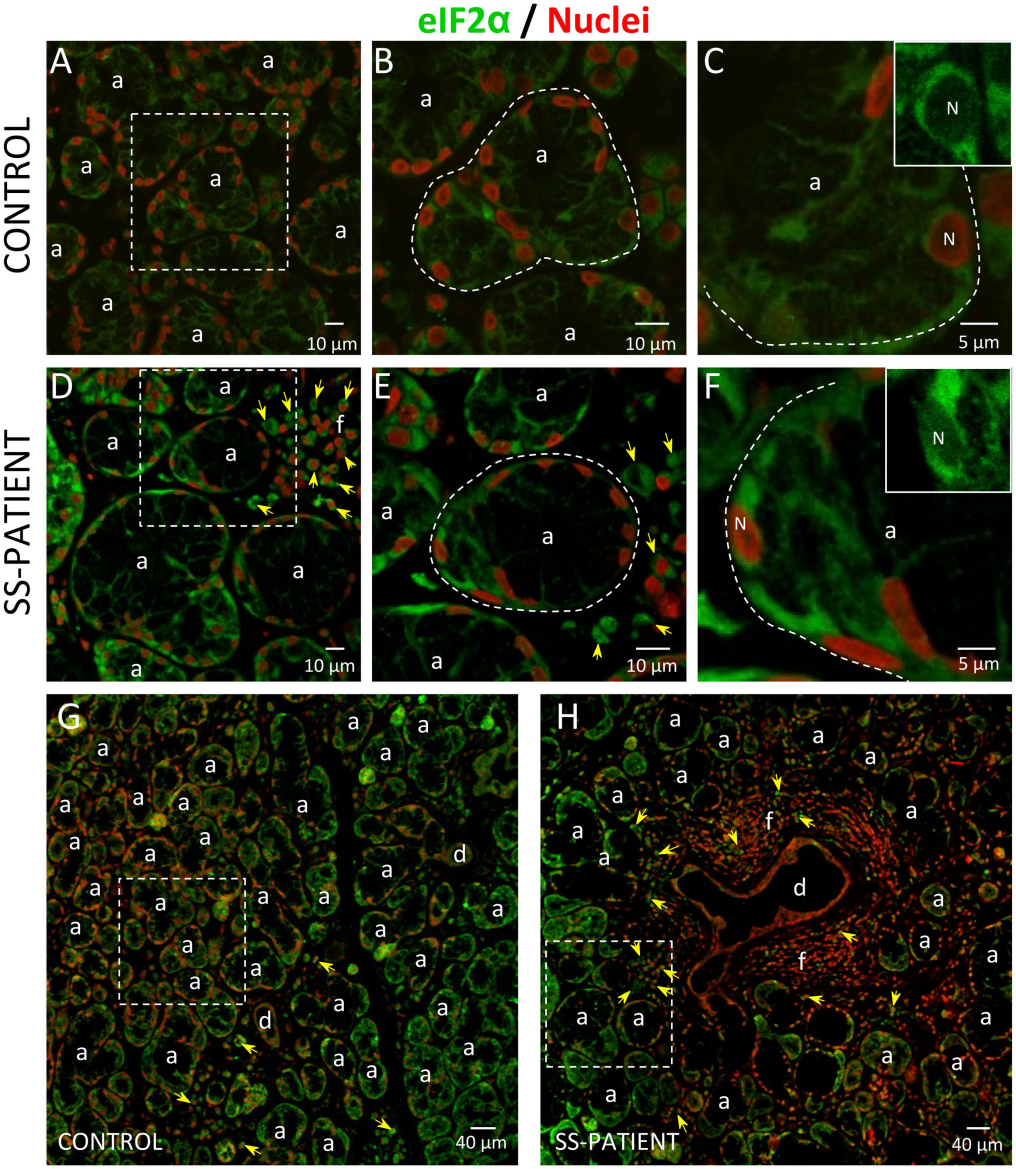
Localization of eIF2α in LSGs from control and SS-patients. **(A–C,G)** eIF2α (green) staining was mainly observed in the basolateral cytoplasm of epithelial cells in LSGs from control subjects. **(D–F,H)** eIF2α (green) staining was also observed in the basolateral cytoplasm of epithelial cells and plasma cells (yellow arrows) in LSGs from SS-patients. **(A,D)** Higher magnifications of regions surrounded by broken lines in G and H, respectively. **(B,C,E,F)** Higher magnifications of regions surrounded by broken lines in A and B, respectively. Nuclei (N, red) were counterstained with Hoechst-33342. a: acini; d: duct; f: focus of inflammatory cells. Bars A, B, D and E: 10 μm; C and F: 5 μm; G and H: 40 μm.

**Figure 10:**
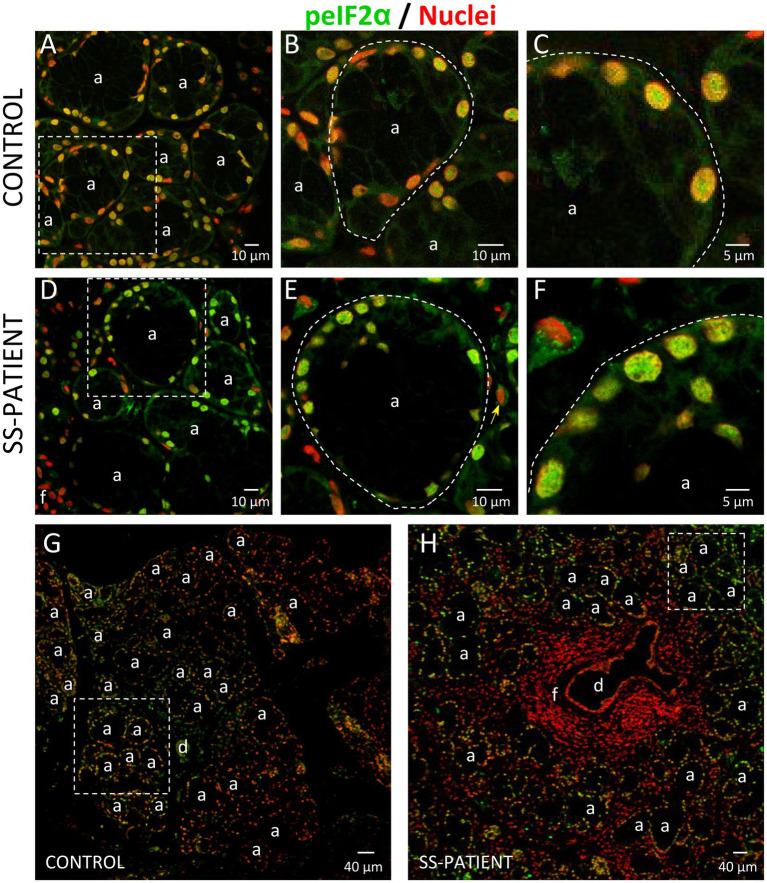
Localization of p-eIF2α in LSGs from controls and SS-patients. **(A–C,G)** p-eIF2α (green) staining was mainly observed in the nuclei of epithelial cells in LSGs from control subjects. **(D–F,H)** stronger p-eIF2α (green) staining was observed in the nuclei of epithelial cells in LSGs from SS-patients. **(A,D)** Higher magnifications of regions surrounded by broken lines in G and H, respectively. **(B,C,E,F)** Higher magnifications of regions surrounded by broken lines in A and B, respectively. Nuclei (red) were counterstained with Hoechst-33342. a: acini; d: duct; f: focus of inflammatory cells. Bars A, B, D and E: 10 μm; C and F: 5 μm; G and H: 40 μm.

The ISR signaling pathways activated by different stressor stimuli converge in the phosphorylation of serine 51 of eIF2α (18). eIF2α transcript levels showed no differences between SS-patients and controls (Figure 8A); meanwhile, eIF2α protein levels significantly increased in SS-patients (*p* = 0.0307) (Figures 8B,C). This observation was repeated for p-eIF2α levels, which also significantly increased (*p* = 0.0144) (Figures 8B,D). The p-eIF2α/eIF2α ratio was unchanged between SS-patients and controls (Figure 8E) and p-eIF2α levels positively correlated with eIF2α (Figure 8F), p-PKR, and PKR protein levels (Supplementary Table S4). The localization of eIF2α by immunofluorescence was mainly found in the cytoplasm of acinar and plasma cells and very weakly in duct cells (Figure 9H). While p-eIF2α was mainly localized in the nuclei of acinar cells (Figure 10), and staining was stronger in LSGs of SS-patients (Figures 10D–F,H). No staining for p-eIF2α was observed in most inflammatory or duct cells (Figure 10H). These results showed that eIF2α protein levels are increased in SS-patients and the increased p-eIF2α levels suggest that ISR activation increases in acinar cells of LSGs from SS-patients.

**Figure 11:**
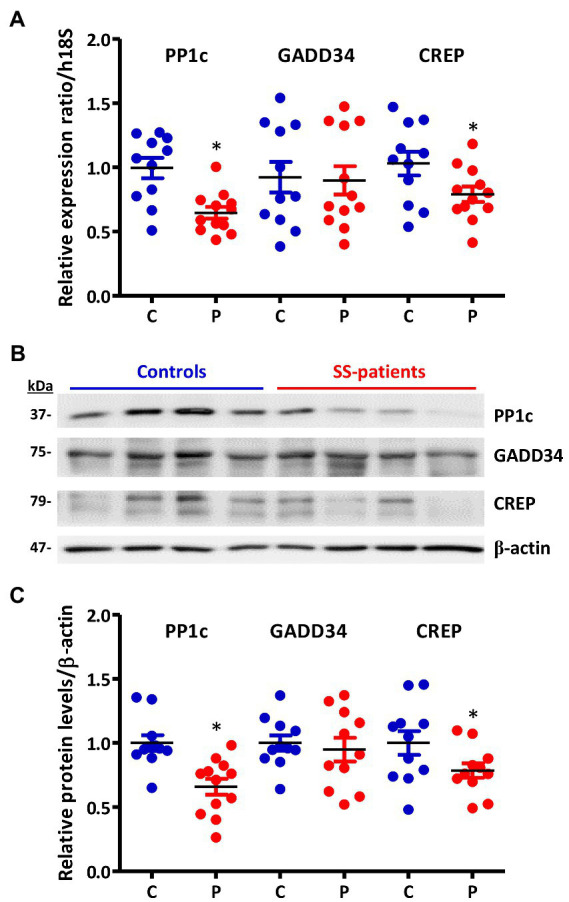
Expression of PP1c, GADD34, and CREP in LSGs from control and SS-patients. **(A)** Dot plot showing PP1c, GADD34, and CREP transcript levels relative to h18S from control (C) and SS-patients (P) (*n* = 11C, 12P). **(B)** Representative images of PP1c, GADD34, and CREP immunoblots from control and SS-patients using β-actin as a loading control. **(C)** Dot plot showing densitometric analysis of PP1c (*n* = 11C y 12P), GADD34 (*n* = 11C, 11P), and CREP (*n* = 11C, 11P). These experiments were repeated at least three times (*). *p* values lower than 0.05 were considered significant.

eIF2α dephosphorylation modulates the ISR and is mediated by the Protein Phosphatase 1 (PP1) complex, which is constituted by the PP1c subunit and one of its two regulatory subunits: GADD34 or CREP (18). In the LSG from SS-patients, PP1c transcripts and protein levels significantly decrease (*p* = 0.0021 and *p* = 0.0008, respectively) (Figures 11A–C). Furthermore, PP1c levels directly correlated with the UWSF and inversely correlated with the presence of Ro, La, ANA autoantibodies, focus score, ESSDAI, and p-PERK/PERK ratio (Supplementary Table S4). GADD34 mRNA and protein levels did not differ significantly between groups (*p* = 0.49 and *p* = 0.29, respectively) (Figures 11A–C). CREP mRNA and protein levels significantly decreased in LSGs from SS-patients (*p* = 0.035 and *p* = 0.044, respectively) (Figures 11A–C). Furthermore, CREP protein levels directly correlated with UWSF and inversely correlated with RF and focus score (Supplementary Table S4). The decrease in components of the PP1c/CREP phosphatase complex suggests permanent activation of the ISR since activated eIF2α would remain phosphorylated.

### 3.3. Increased protein levels of ATF4 and ATF4-target genes related to the antioxidant response in LSGs of SS-patients

**Figure 12:**
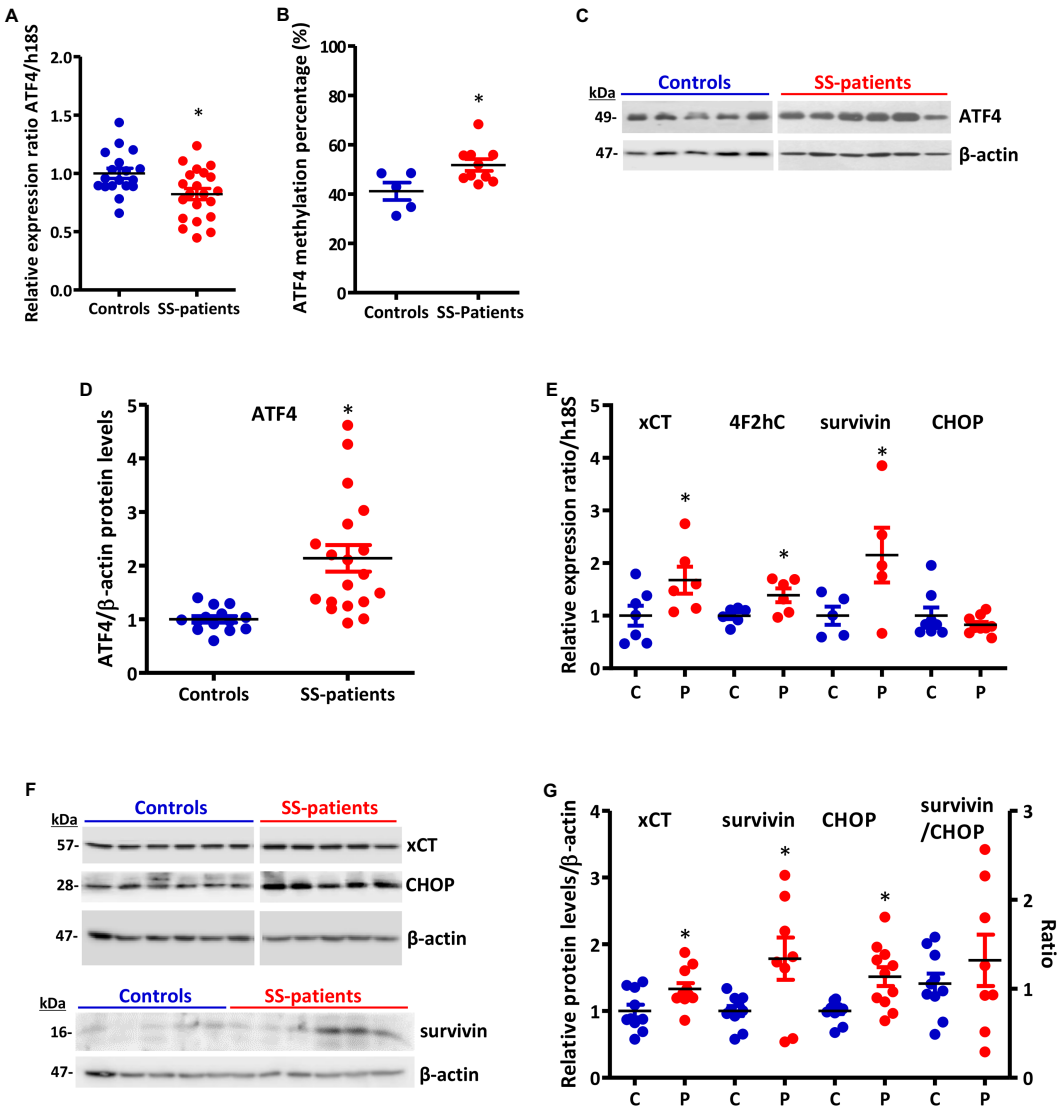
Expression of ATF4 and ATF4-target genes in LSGs of control and SS-patients. **(A)** Dot plot showing ATF4 transcript levels relative to h18S from control (C) and SS-patients (P) (*n* = 18C, 21P). **(B)** Dot plot showing ATF4 promoter-specific DNA methylation (*n* = 5C, 10P). **(C)** representative images of ATF4 immunoblot from control and SS-patients using β-actin as a loading control. **(D)** Dot plot showing densitometric analysis of ATF4 (*n* = 11C, 19P). **(E)** Dot plot showing xCT (*n* = 7C, 6P), 4F2hc (*n* = 6C, 6P), survivin (*n* = 5C, 5P) and CHOP (*n* = 8C, 9P) transcript levels relative to h18S. **(F)** Representative images of xCT, survivin, and CHOP immunoblot from control and SS-patients using β-actin as a loading control. **(G)** Dot plot showing densitometric analysis of xCT (*n* = 10C, 11P), survivin (*n* = 10C, 8P), CHOP (*n* = 11C, 11P) and survivin/CHOP ratio (*n* = 10C, 8P). These experiments were repeated at least three times (*). *p* values lower than 0.05 were considered significant.

**Figure 13:**
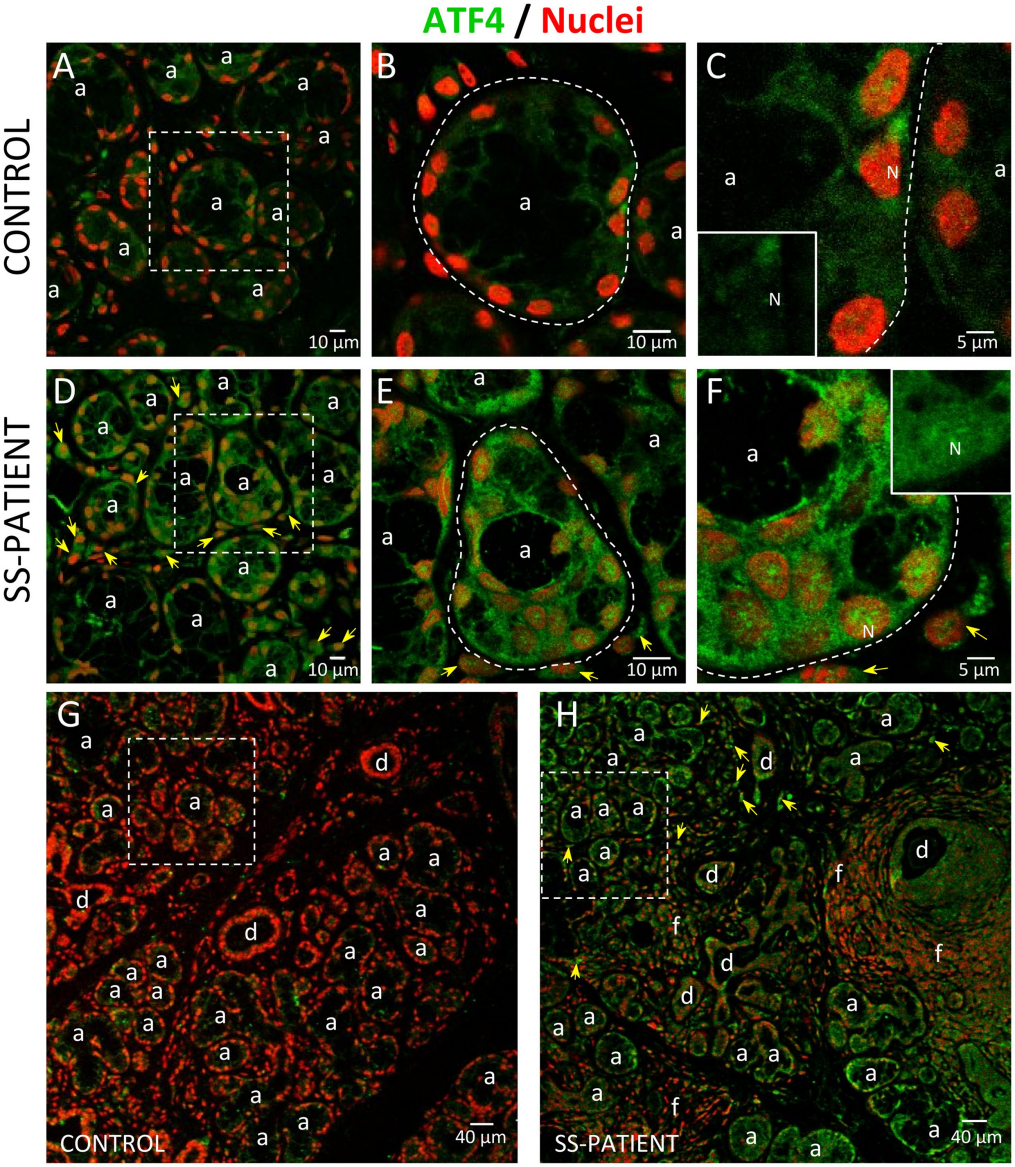
Localization of ATF4 in LSGs from control and SS-patients. **(A–C,G)** ATF4 (green) shows weak staining in the cytoplasm of epithelial cells in LSGs from control subjects. **(D–F,H)** Stronger ATF4 (green) staining was observed in the cytoplasm and nuclei (N) of epithelial cells in LSGs from SS-patients and in plasma cells (yellow arrows). **(A,D)** higher magnifications of regions surrounded by broken lines in G and H, respectively. **(B,C,E,F)** Higher magnifications of regions surrounded by broken lines in A and B, respectively. Nuclei (red) were counterstained with Hoechst-33342. a: acini; d: duct; f: focus of inflammatory cells. Bars A, B, D and E: 10 μm; C and F: 5 μm; G and H: 40 μm.

Since ATF4 is a common downstream target that integrates the signaling pathways of all four eIF2α kinases, the p-eIF2α/ATF4 pathway is responsible for inducing ISR activated genes (17). Although p-eIF2α induces the preferential translation of ATF4 mRNA, as a first approximation, we evaluated ATF4 mRNA levels, finding decreased levels in the LSGs of SS-patients (*p* = 0.019), concomitant with hypermethylation of the ATF4 promoter (*p* = 0.038) (Figures 12A,B, respectively), and an inverse correlation between them (Supplementary Table S4). This result indicates that ATF4 mRNA levels could be regulated by the methylation state of its promoter. As previously mentioned, eIF2α phosphorylation not only inhibits global translation but also promotes translation of ATF4 mRNA by its upstream open reading frames (uORFs) (43), which is related to the transcription of genes involved in inflammation and cellular adaptability or apoptosis in response to stress (42). SS-patients presented a significant increase in ATF4 protein levels (*p* < 0.0001) (Figures 12C,D). Moreover, the immunofluorescence results corroborated the increase in ATF4 protein levels, showing a stronger ATF4 staining in acinar cells from the LSGs of SS-patients (Figure 13). ATF4 staining was mainly located in the cytoplasm and nucleus of acinar cells (Figures 13A–F) and was also detected in plasma and duct cells (Figures 13D,H). ATF4 protein levels showed a positive correlation with clinical parameters, including ESSDAI, focus score, and the presence of autoantibodies (Ro, La, ANA, and RF) (Supplementary Table S4).

Relevant targets of the ATF4 transcription factor are components of system Xc^−^, a cystine-glutamate exchanger system (44). This exchanger system is comprised two subunits, 4F2hc stabilizes the protein to the membrane and xCT confers substrate selectivity (45). We observed a significant increase in xCT and 4F2hc mRNA levels (*p* = 0.037 and *p* = 0.047, respectively) in LSGs of SS-patients (Figure 12E). SS-patients also showed a significant increase in xCT protein levels (*p* = 0.031) (Figures 12F,G). The detection of xCT by immunofluorescence showed that xCT is localized in the plasma and acinar cells of LSGs (Supplementary Figure S3), showing a stronger staining in LSGs from SS-patients (Supplementary Figures S3D–F,H). Additionally, xCT protein levels positively correlated with Ro and La autoantibodies, focus score, and inversely correlated with the UWSF (Supplementary Table S4). Since system Xc^−^ participates in GSH production, whose increased levels are associated with an increase in cell survival, system Xc^−^ could participate in regulating the intracellular oxidative environment in the LSGs of SS-patients (46–48).

Previous studies from our laboratory showed a balance in the expression of pro-apoptotic and anti-apoptotic genes in the epithelial cells of LSG of SS-patients (15). Considering the increased ATF4 protein levels and the responses regulated by this factor, we evaluated the expression of the anti-apoptotic molecule survivin, which is regulated by ATF4 through the STAT3 pathway (49), and the pro-apoptotic molecule CHOP, also regulated by ATF4 (50). We observed a significant increase in the mRNA levels of survivin (*p* = 0.028) and no changes in mRNA levels of CHOP (*p* = 0.3) in SS-patients (Figure 12E). Furthermore, the LSGs of SS-patients presented a significant increase in survivin (*p* = 0.034) and CHOP (*p* = 0.0043) protein levels (Figures 12F–G). CHOP staining was almost undetected in LSGs from controls (Supplementary Figures S4A–C,G). Stronger CHOP staining was observed in the nuclei and cytoplasm of acinar, ductal, and inflammatory cells in SS-patients (Supplementary Figures S4D–F,H) in agreement with the increased protein levels observed by Western blot. When we evaluated the ratio between survivin and CHOP, SS-patients and controls showed a balanced expression of both molecules (*p* = 0.38) (Figure 12G).

### 3.4. NRF2 expression in the LSG of SS-patients

**Figure 14:**
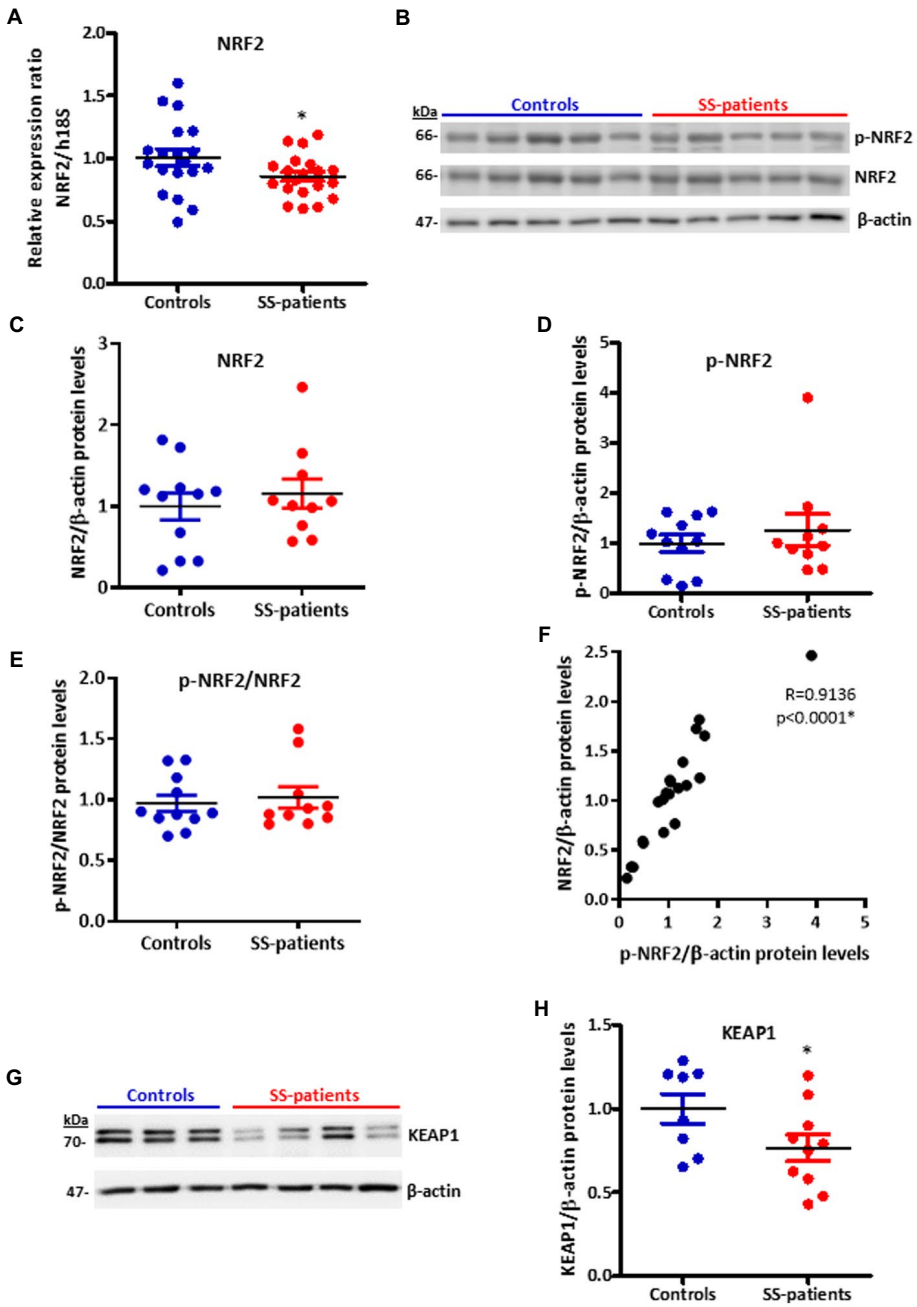
Expression of NRF2, p-NRF2, and KEAP1 in LSGs from control and SS-patients. **(A)** Dot plot showing NRF2 transcript levels relative to h18S from control (C) and SS-patients (P) (*n* = 19C, 20P). **(B)** Representative images of p-NRF2 and NRF2 immunoblots from control and SS-patients using β-actin as a loading control. **(C)** Dot plot showing densitometric analysis of NRF2 (*n* = 11C, 10P). **(D)** Dot plot showing densitometric analysis of p-NRF2 (*n* = 11C, 10P). **(E)** Dot plot showing the p-NRF2/NRF2 ratio (*n* = 11C, 10P). **(F)** Spearman’s correlation between p-NRF2 and NRF2 protein levels. **(G)** Representative images of KEAP1 immunoblots from control and SS-patients using β-actin as a loading control. **(H)** Dot plot showing densitometric analysis of KEAP1. These experiments were repeated at least three times (*). *p* values lower than 0.05 were considered significant.

**Figure 15:**
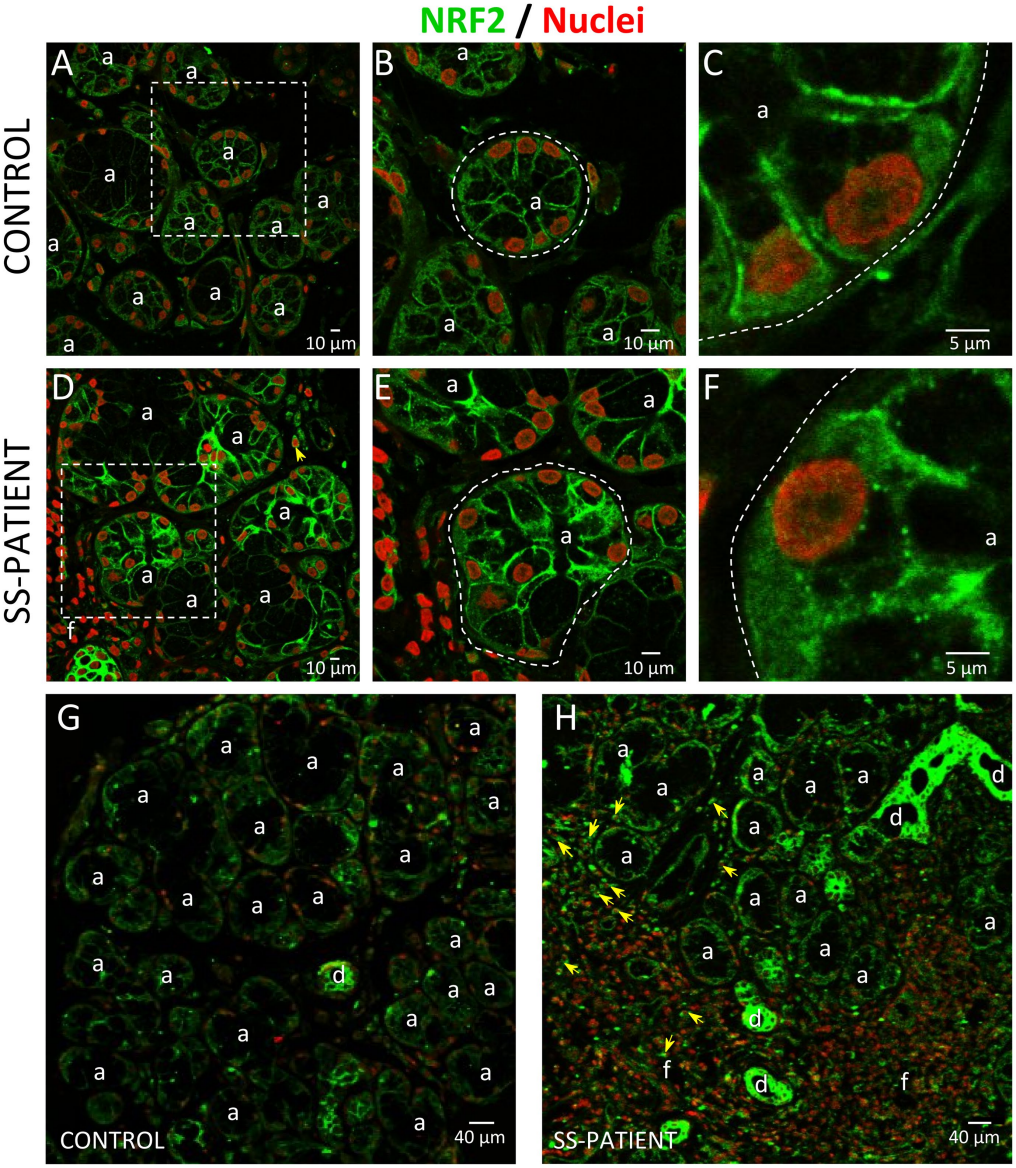
Localization of NRF2 in LSGs from control and SS-patients. **(A–C,G)** NRF2 (green) staining was mainly observed in the basolateral cytoplasm and plasma membrane of epithelial cells in LSGs from control subjects. **(D–F,H)** Stronger NRF2 (green) staining was observed in the basolateral cytoplasm and plasma membrane of epithelial and plasma cells (yellow arrows) in LSGs from SS-patients. **(A,D)** Higher magnifications of regions bounded by broken lines in G and H, respectively. **(B,C,E,F)** Higher magnifications of regions bounded by broken lines in A and B, respectively. Nuclei (red) were counterstained with Hoechst-33342. a: acini; d: duct; f: focus of inflammatory cells. Bars A, B, D, and E: 10 μm; C and F: 5 μm; G and H: 40 μm.

Another key transcription factor in ISR is NRF2, which is phosphorylated by activated PERK (26). Under stress conditions, NRF2 forms heterodimers with ATF4 and induces the expression of ISR target genes (51) and ATF4 itself (52). LSGs from SS-patients showed significant decreases in NRF2 mRNA levels (*p* = 0.03) (Figure 14A). There were no differences in the protein levels of NRF2 (*p* = 0.50), p-NRF2 (*p* = 0.50), and the p-NRF2/NRF2 ratio (Figures 14B–E). p-NRF2 protein levels strongly correlated with total NRF2 protein levels (Figure 14F). Additionally, KEAP1 protein levels, an inhibitor of NRF2 that sequesters NRF2 in the cytosol preventing it from entering the nucleus and acting as a transcription factor (27), significantly decreased in the LSGs of SS-patients (*p* = 0.033, Figures 14G,H). NRF2 was mainly observed in the basolateral cytoplasm and toward the cell boundary of acinar and duct cells of LSGs and in plasma cells (Figure 15). Although there were no differences in NRF2 levels detected by Western blot, a strong staining was observed in acinar and duct cell of LSGs from SS-patients. Based on the obtained data, NRF2 could have a contribution to the ISR in the LSGs of SS-patients.

**Figure 16:**
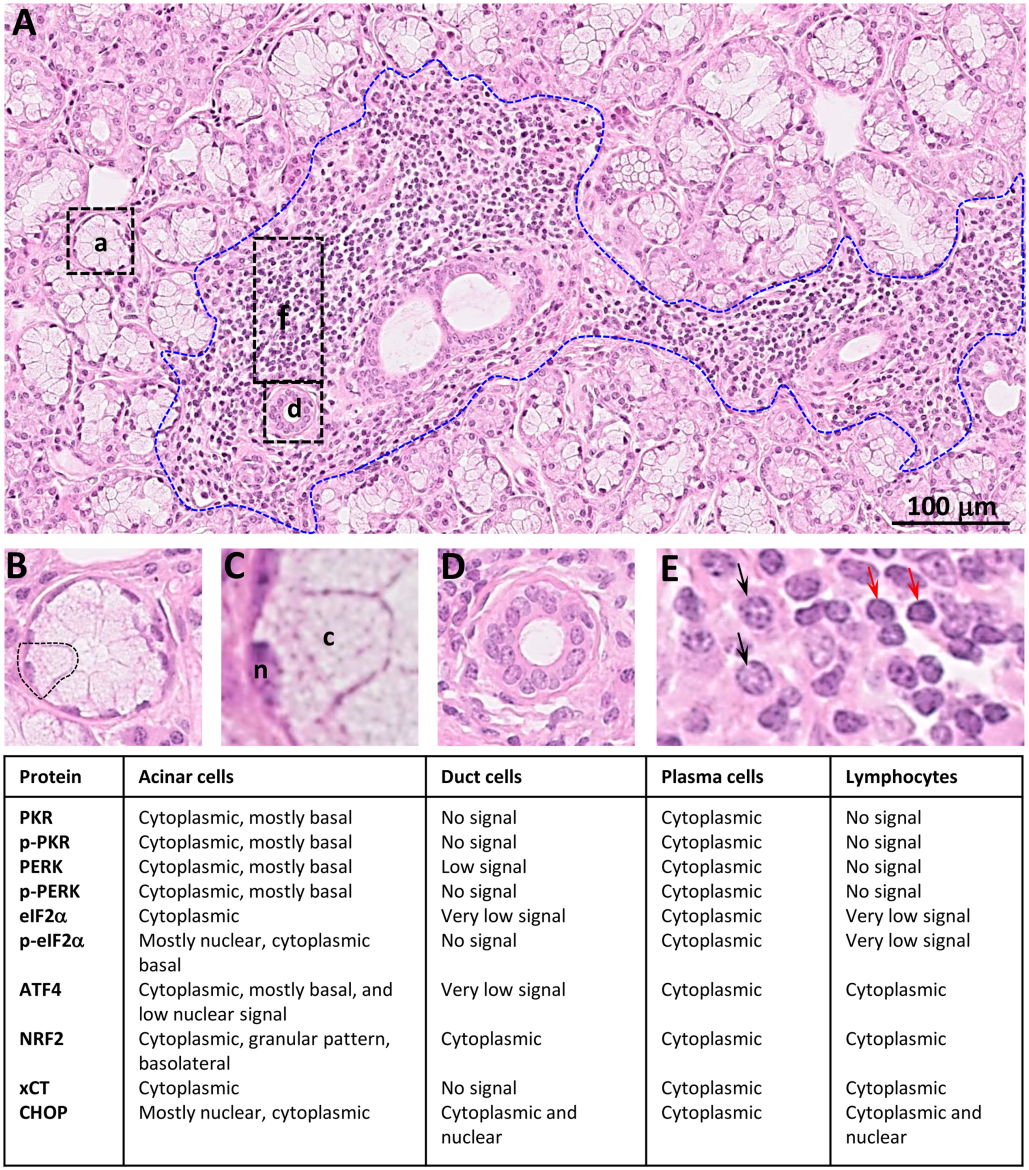
Summary of distribution observed for ISR proteins in LSGs from SS-patients. **(A)** Histology of a LSG from a representative SS-patient showing several acini (a), few ducts (d), and a lymphoplasmacytic focus (f, bounded by blue broken line). Bar: 100 μm. **(B)** Higher magnifications of an acini. **(C)** Higher magnifications of an acinar cell, n: nuclei; c: cytoplasm. **(D)** Higher magnification of a duct. **(E)** Higher magnification of inflammatory cells showing plasma cells (black arrows) and lymphocytes (red arrows).

Figure 16 summarizes the distribution of the ISR components observed in LSG from SS-patients.

## 4. Discussion

In this study, the increased levels of PKR, p-PKR, PERK, p-PERK/PERK ratio, eIF2α, p-eIF2α, ATF4, and ATF4-target genes such as xCT, survivin and CHOP determined by Western blot, as well as staining by immunofluorescence, indicated increased activation of the ISR in acinar cells of LSGs from SS-patients. In these glands, plasma cells also show strong staining for ISR components such as PKR, p-PKR, PERK, eIF2α, ATF4, NRF2, xCT, and CHOP. This is not surprising since the high antibody production rate induces the expansion of the secretory machinery, triggering compensatory stress responses in plasma cells (53).

Increased PKR mRNA and protein levels, and p-PKR protein levels were observed in LSGs of SS-patients, indicating higher activation of this sensor. PKR activation in the LSG of SS-patients is in tune with the high levels of oxidative stress (13), ER stress (24), and pro-inflammatory cytokines (54). In addition, increased PKR activation in LSGs of SS-patients could be associated to viral infections, such as chronic or recurrent infection of epithelial cells of SS-patients with Epstein–Barr virus (55) and coxsackievirus (56). Interestingly, interferons such as IFN-α, IFN-β, and IFN-γ could be activating agents of the PKR sensor in SS, because these cytokines are elevated in LSGs from SS-patients, and SS is frequently considered an interferonopathy (57, 58). Moreover, PKR binds to various RNAs, such as retrotransposons, satellite RNAs and mitochondrial RNAs (mtRNAs), which can regulate PKR phosphorylation and signaling, especially under stress (59). It is worthy to note that severe ultrastructural alterations of mitochondria have been observed in salivary gland cells from SS-patients (60).

There are currently no published results in SS-patients concerning PERK, one of the four eIF2α kinases involved in ISR activation and also a member of UPR. Our previous studies on the UPR in LSGs of SS-patients show attenuation of the IRE1α pathway and increased activation of the ATF6α pathway (16, 25). Here, we reported increased activation of the PERK pathway. While we observed decreased PERK protein levels, the p-PERK/PERK ratio increased, and remarkably stronger p-PERK staining was observed in epithelial cells, indicating that this sensor is more active in LSGs from SS-patients. The differential behavior in the time course-kinetics of UPR sensor activation observed in SS-patients was described in other tissues under persistent ER stress (61). Additionally, there is a link between inflammation and ER stress in chronic inflammatory pathologies such as ulcerative colitis (62), suggesting that the chronic inflammatory environment and the loss of cellular homeostasis described in SS-patients could modulate ER stress conditions in LSGs.

Increased eIF2α protein levels are accompanied by high phosphorylation levels of this factor mainly observed in the nuclei of acinar cells. However, other studies have also described p-eIF2α in the nucleus, which would be mediated by the presence of a nuclear localization signal and a nuclear export signal (63). Interestingly, these signals are exposed in the phosphorylated form of the protein (63), explaining why only p-eIF2α is seen in the nucleus in our study. In summary, the results observed for eIF2α suggest that in SS-patients global protein synthesis is reduced in response to increased cellular stress. However, the translation of some specific mRNAs such as ATF4 is induced.

SS-patients showed high ATF4 protein levels but low mRNA levels. One possible explanation could be the methylation state of the ATF4 promoter since SS-patients presented high methylation levels. ATF4 mRNA levels correlate with the methylation percentage of its promoter, similarly to what was previously observed in our laboratory for IRE1α and XBP-1 promoters in LSGs from SS-patients (25).

Although the mRNA encoding ATF4 is present at low levels in LSGs from SS-patients, translation of ATF4 increases under conditions of cellular stress through a regulatory 5′ leader sequence with multiple uORFs, while the global protein synthesis rate decreases. The ATF4 transcription factor regulates the expression of genes involved in the UPR, amino acid metabolism, redox reactions, cell survival, and apoptosis (46). Together, the positive correlation between ATF4 protein levels and clinical parameters associated with disease activity, increased expression of ATF4 targets, such as system Xc^−^ components (64), and the balanced expression of anti/pro-apoptotic proteins (15), suggest ISR activation as a potential adaptive mechanism of LSGs from SS-patients.

The increased activation of kinases (PKR and PERK) that phosphorylate eIF2α and the decreased expression of phosphatase complex components that dephosphorylate eIF2α to terminate the ISR, such as PP1c and CREP, indicate that the ISR remains active in LSGs from SS patients. This would explain the high ATF4 protein levels observed by Western blot and immunofluorescence, as well as the increased expression of target genes involved in the antioxidant response, such as xCT and 4F2hc. Previous studies indicated that inflammation or alteration of ER calcium levels in LSGs from SS-patients (9, 65) induce oxidative stress conditions, generating high concentrations of reactive oxygen species, and severe cellular damage (13). The system Xc^−^ could reduce oxidative stress by mediating cystine transport into the cell in exchange for glutamate (44). Cystine regulates GSH cellular levels, the main endogenous intracellular antioxidant, protecting cells against oxidative stress (66). Neuronal cell lines selected for resistance against oxidative stress, presented xCT upregulation induced by ATF4, indicating that ATF4 has a central role in protecting cells under oxidative damage and promoting cell survival (44). The positive correlation between xCT with clinical parameters suggests that system Xc^−^ could be part of a compensatory response seeking to alleviate a stressful condition developed in LSGs of SS-patients. Furthermore, the high xCT levels observed in SS-patients with increased ATF4 protein levels support previous data demonstrating that ATF4 regulates xCT expression and indicates that ATF4 modulates adaptive mechanisms in LSGs from SS-patients (44). xCT expression is not only induced by ATF4 but also by NRF2 (67), which show no significant differences in total protein extracts but a stronger staining intensity in epithelial cells of LSGs from SS-patients.

Other ATF4 target genes evaluated in this study were survivin and CHOP (61). Survivin (BIRC5) inhibits apoptosis, and its overexpression is associated with autoimmunity. It promotes autoreactive lymphocytes in some immune tissues during imbalanced expression of pro-apoptotic proteins (68). In this study, we observed increased survivin mRNA levels. The survivin promoter contains ATF4 response elements, supporting the idea that ATF4 acts as an inducer of survivin expression. Although ATF4 also regulates CHOP transcription (50), we did not observe increased mRNA levels in LSGs of SS-patients, but we found increased protein levels. Under cellular stress conditions, preferential translation of CHOP occurs by a mechanism involving a single uORF, which is in the 5′-leader of its mRNA (69). Our results showed balanced survivin and CHOP protein levels in the LSGs of control and SS-patients. This observation is in line with previous data showing balanced expression of anti- and pro-apoptotic molecules by a microarray assay (15), low levels of active capase-3 (16), low levels of TUNEL positive nuclei (14), and increased ER-associated protein degradation (ERAD) activation *via* ATF6α (16). Together, these results suggest the existence of compensatory mechanisms that allow cells to survive despite diverse alterations and the ISR could act as another cell survival mechanism in LSGs from SS-patients.

NRF2 is another key transcription factor in ISR, and it is phosphorylated by PERK, participating in cell survival processes against ER and oxidative stress conditions (42). Our results showed decreased NRF2 mRNA without significant differences in protein and phosphorylated protein levels. However, immunofluorescence results revealed the valuable importance of assessing the localization, showing higher staining in acinar cells of SS-patients, suggesting that NRF2 could be induced in response to oxidative stress previously reported in the LSG of SS-patients (13).

NRF2 abundance in cells is tightly controlled by ubiquitination and degradation in the proteasome (27). KEAP1 is very important in this process as it sequesters NRF2 in the cytosol, preventing it from translocating to the nucleus and acting as a transcription factor. KEAP1 also acts as an adapter protein that allows NRF2 to interact with E3 ubiquitin ligase complexes so that NRF2 is ubiquitinated and degraded (27). In this study, we found decreased KEAP1 protein levels in the LSGs of SS-patients compared to controls, which could be another compensatory mechanism in response to oxidative stress. The E3-ubiquitin ligase complex that primarily participates in NRF2 degradation depends on cullin-3 (Cul3), a core component and scaffold protein of the E3 ligase complex, which is one of the most increased in SS-patients, together with other cullins (cullin-5 and cullin-1) (15). In summary, the results of NRF2 immunofluorescence and the KEAP1 protein levels suggest that NRF2 could contribute to the ISR in the LSGs of SS-patients.

In conclusion, the results of this study showed that there is increased ISR activation in LSGs of SS-patients. Since ATF4 regulates the expression of genes involved in adaptive responses to cellular stress, such as system Xc^−^ components, its increased expression could be part of a rescue response against the various stressful conditions to which the LSGs of SS-patients are subjected (viruses, cytokines, oxidative stress, and ER stress) promoting cell survival.

## Data availability statement

The original contributions presented in the study are included in the article/Supplementary material, further inquiries can be directed to the corresponding authors.

## Ethics statement

The studies involving human participants were reviewed and approved by The Ethical Committee of the Faculty of Medicine, University of Chile approved this study (N° 001-2021). The patients/participants provided their written informed consent to participate in this study.

## Author contributions

PC, VB, DJ, IC, M-JB, and M-JG conceived the study and conceived and designed the experiments, while PC, VB, DJ, IC, SM, SA, CM, SG, MH, M-JB, and M-JG performed them. SA, SG, and CM were involved in clinical data collection. PC, VB, DJ, M-JB, and M-JG wrote the manuscript. All authors contributed to the article and approved the submitted version.

## Funding

This study was financially supported by Fondecyt-Chile (#1210055 to M-JG, SA, CM, SG, IC, M-JB); Enlace-VID Universidad de Chile (ENL04/20 to M-JG); Fondecyt-Iniciación (11201058 to M-JB); Centro Ciencia & Vida, (FB210008), Financiamiento Basal para Centros Científicos y Tecnológicos de Excelencia de ANID; PhD fellowship Conicyt-Chile to PC and DJ; and FONDEQUIP EQM170098. Líneas de apoyo a la investigación financiadas por el ICBM (2022).

## Conflict of interest

The authors declare that the research was conducted in the absence of any commercial or financial relationships that could be construed as a potential conflict of interest.

## Publisher’s note

All claims expressed in this article are solely those of the authors and do not necessarily represent those of their affiliated organizations, or those of the publisher, the editors and the reviewers. Any product that may be evaluated in this article, or claim that may be made by its manufacturer, is not guaranteed or endorsed by the publisher.

## References

[1] Mavragani CP Moutsopoulos HM. Sjögren's syndrome: old and new therapeutic targets. J Autoimmun. (2020) 110:102364. doi: 10.1016/j.jaut.2019.102364, PMID: 31831255

[2] Castle JD Arvan P Cameron R. Protein production and secretion in exocrine cells. J Dent Res. (1987) 66:633–7. doi: 10.1177/00220345870660s105 3476628

[3] Thornton DJ Rousseau K McGuckin MA. Structure and function of the polymeric mucins in airways mucus. Annu Rev Physiol. (2008) 70:459–86. doi: 10.1146/annurev.physiol.70.113006.100702, PMID: 17850213

[4] Castro I Albornoz N Aguilera S Barrera MJ González S Núñez M. Aberrant MUC1 accumulation in salivary glands of Sjögren's syndrome patients is reversed by TUDCA in vitro. Rheumatology (Oxford). (2020) 59:742–53. doi: 10.1093/rheumatology/kez316, PMID: 31377809

[5] Sung HH Castro I González S Aguilera S Smorodinsky NI Quest A. MUC1/SEC and MUC1/Y overexpression is associated with inflammation in Sjögren's syndrome. Oral Dis. (2015) 21:730–8. doi: 10.1111/odi.12339, PMID: 25757505

[6] Alliende C Kwon YJ Brito M Molina C Aguilera S Pérez P. Reduced sulfation of muc5b is linked to xerostomia in patients with Sjögren syndrome. Ann Rheum Dis. (2008) 67:1480–7. doi: 10.1136/ard.2007.078246, PMID: 17998215

[7] Castro I Aguilera S Brockhausen I Alliende C Quest AF Molina C. Decreased salivary sulphotransferase activity correlated with inflammation and autoimmunity parameters in Sjogren's syndrome patients. Rheumatology (Oxford). (2012) 51:482–90. doi: 10.1093/rheumatology/ker351, PMID: 22101162

[8] Chaudhury NM Proctor GB Karlsson NG Carpenter GH Flowers SA. Reduced Mucin-7 (Muc7) sialylation and altered saliva rheology in Sjögren's syndrome associated Oral dryness. Mol Cell Proteomics. (2016) 15:1048–59. doi: 10.1074/mcp.M115.052993, PMID: 26631508 PMC4813687

[9] Enger TB Aure MH Jensen JL Galtung HK. Calcium signaling and cell volume regulation are altered in Sjögren's syndrome. Acta Odontol Scand. (2014) 72:549–56. doi: 10.3109/00016357.2013.879995, PMID: 24471729

[10] Goicovich E Molina C Pérez P Aguilera S Fernández J Olea N. Enhanced degradation of proteins of the basal lamina and stroma by matrix metalloproteinases from the salivary glands of Sjögren's syndrome patients: correlation with reduced structural integrity of acini and ducts. Arthritis Rheum. (2003) 48:2573–84. doi: 10.1002/art.11178, PMID: 13130477

[11] Fox PC Brennan M Di Sun P. Cytokine expression in human labial minor salivary gland epithelial cells in health and disease. Arch Oral Biol. (1999) 44:S49–52. doi: 10.1016/S0003-9969(99)90018-3, PMID: 10414856

[12] Chong WC Shastri MD Eri R. Endoplasmic reticulum stress and oxidative stress: a vicious nexus implicated in bowel disease pathophysiology. Int J Mol Sci. (2017) 18:771. doi: 10.3390/ijms18040771, PMID: 28379196 PMC5412355

[13] Kurimoto C Kawano S Tsuji G Hatachi S Jikimoto T Sugiyama D. Thioredoxin may exert a protective effect against tissue damage caused by oxidative stress in salivary glands of patients with Sjögren's syndrome. J Rheumatol. (2007) 34:2035–43. 17896802

[14] Ohlsson M Skarstein K Bolstad AI Johannessen AC Jonsson R. Fas-induced apoptosis is a rare event in Sjögren's syndrome. Lab Investig. (2001) 81:95–105. doi: 10.1038/labinvest.3780215, PMID: 11204278

[15] Pérez P Anaya JM Aguilera S Urzúa U Munroe D Molina C. Gene expression and chromosomal location for susceptibility to Sjögren's syndrome. J Autoimmun. (2009) 33:99–108. doi: 10.1016/j.jaut.2009.05.001, PMID: 19523788

[16] Barrera MJ Aguilera S Castro I Cortés J Bahamondes V Quest AF. Pro-inflammatory cytokines enhance ERAD and ATF6α pathway activity in salivary glands of Sjögren's syndrome patients. J Autoimmun. (2016) 75:68–81. doi: 10.1016/j.jaut.2016.07.006, PMID: 27461470

[17] Costa-Mattioli M Walter P. The integrated stress response: from mechanism to disease. Science. (2020) 368:eaat5314. doi: 10.1126/science.aat5314, PMID: 32327570 PMC8997189

[18] Pakos-Zebrucka K Koryga I Mnich K Ljujic M Samali A Gorman AM. The integrated stress response. EMBO Rep. (2016) 17:1374–95. doi: 10.15252/embr.201642195, PMID: 27629041 PMC5048378

[19] Shimazawa M Hara H. Inhibitor of double stranded RNA-dependent protein kinase protects against cell damage induced by ER stress. Neurosci Lett. (2006) 409:192–5. doi: 10.1016/j.neulet.2006.09.074, PMID: 17055645

[20] Kang R Tang D. PKR-dependent inflammatory signals. Sci Signal. (2012) 5:pe47. doi: 10.1126/scisignal.2003511, PMID: 23092889 PMC3656404

[21] Han AP Yu C Lu L Fujiwara Y Browne C Chin G. Heme-regulated eIF2alpha kinase (HRI) is required for translational regulation and survival of erythroid precursors in iron deficiency. EMBO J. (2001) 20:6909–18. doi: 10.1093/emboj/20.23.6909, PMID: 11726526 PMC125753

[22] Kimball SR. Regulation of translation initiation by amino acids in eukaryotic cells. Prog Mol Subcell Biol. (2001) 26:155–84. doi: 10.1007/978-3-642-56688-2_6 11575165

[23] Gonen N Sabath N Burge CB Shalgi R. Widespread PERK-dependent repression of ER targets in response to ER stress. Sci Rep. (2019) 9:4330. doi: 10.1038/s41598-019-38705-5, PMID: 30867432 PMC6416471

[24] Barrera MJ Aguilera S Castro I González S Carvajal P Molina C. Endoplasmic reticulum stress in autoimmune diseases: can altered protein quality control and/or unfolded protein response contribute to autoimmunity? A critical review on Sjögren's syndrome. Autoimmun Rev. (2018) 17:796–808. doi: 10.1016/j.autrev.2018.02.009, PMID: 29890347

[25] Sepúlveda D Barrera MJ Castro I Aguilera S Carvajal P Lagos C. Impaired IRE1α/XBP-1 pathway associated to DNA methylation might contribute to salivary gland dysfunction in Sjögren's syndrome patients. Rheumatology (Oxford). (2018) 57:1021–32. doi: 10.1093/rheumatology/key021, PMID: 29534223

[26] Cullinan SB Zhang D Hannink M Arvisais E Kaufman RJ Diehl JA. Nrf2 is a direct PERK substrate and effector of PERK-dependent cell survival. Mol Cell Biol. (2003) 23:7198–209. doi: 10.1128/MCB.23.20.7198-7209.2003, PMID: 14517290 PMC230321

[27] He F Ru X Wen T. NRF2, a transcription factor for stress response and beyond. Int J Mol Sci. (2020) 21:4777. doi: 10.3390/ijms21134777, PMID: 32640524 PMC7369905

[28] Wortel IMN van der Meer LT Kilberg MS van Leeuwen FN. Surviving stress: modulation of ATF4-mediated stress responses in Normal and malignant cells. Trends Endocrinol Metab. (2017) 28:794–806. doi: 10.1016/j.tem.2017.07.003, PMID: 28797581 PMC5951684

[29] Seo HY Jang BK Jung YA Lee EJ Kim HS Jeon JH. Phospholipase D1 decreases type I collagen levels in hepatic stellate cells via induction of autophagy. Biochem Biophys Res Commun. (2014) 449:38–43. doi: 10.1016/j.bbrc.2014.04.149, PMID: 24802400

[30] Chen Y Wang JJ Li J Hosoya KI Ratan R Townes T. Activating transcription factor 4 mediates hyperglycaemia-induced endothelial inflammation and retinal vascular leakage through activation of STAT3 in a mouse model of type 1 diabetes. Diabetologia. (2012) 55:2533–45. doi: 10.1007/s00125-012-2594-1, PMID: 22660795 PMC3412945

[31] Bonnet MC Daurat C Ottone C Meurs EF. The N-terminus of PKR is responsible for the activation of the NF-kappaB signaling pathway by interacting with the IKK complex. Cell Signal. (2006) 18:1865–75. doi: 10.1016/j.cellsig.2006.02.010, PMID: 16600570

[32] Jiang Z Zamanian-Daryoush M Nie H Silva AM Williams BR Li X. Poly(I-C)-induced toll-like receptor 3 (TLR3)-mediated activation of NFkappa B and MAP kinase is through an interleukin-1 receptor-associated kinase (IRAK)-independent pathway employing the signaling components TLR3-TRAF6-TAK1-TAB2-PKR. J Biol Chem. (2003) 278:16713–9. doi: 10.1074/jbc.M300562200, PMID: 12609980

[33] Dabo S Maillard P Collados Rodriguez M Hansen MD Mazouz S Bigot DJ. Meurs: inhibition of the inflammatory response to stress by targeting interaction between PKR and its cellular activator PACT. Sci Rep. (2017) 7:16129. doi: 10.1038/s41598-017-16089-8, PMID: 29170442 PMC5701060

[34] Onat UI Yildirim AD Tufanli Ö Çimen I Kocatürk B Veli Z. Intercepting the lipid-induced integrated stress response reduces atherosclerosis. J Am Coll Cardiol. (2019) 73:1149–69. doi: 10.1016/j.jacc.2018.12.055, PMID: 30871699 PMC6424590

[35] Shiboski CH Shiboski SC Seror R Criswell LA Labetoulle M Lietman TM. Group: 2016 American College of Rheumatology/European league against rheumatism classification criteria for primary Sjögren's syndrome: a consensus and data-driven methodology involving three international patient cohorts. Ann Rheum Dis. (2017) 76:9–16. doi: 10.1136/annrheumdis-2016-210571, PMID: 27789466

[36] Daniels TE. Labial salivary gland biopsy in Sjögren's syndrome. Assessment as a diagnostic criterion in 362 suspected cases. Arthritis Rheum. (1984) 27:147–56. doi: 10.1002/art.1780270205 6696772

[37] Kwon YJ Pérez P Aguilera S Molina C Leyton L Alliende C. Involvement of specific laminins and nidogens in the active remodeling of the basal lamina of labial salivary glands from patients with Sjögren's syndrome. Arthritis Rheum. (2006) 54:3465–75. doi: 10.1002/art.22177, PMID: 17075843

[38] Pfaffl MW Horgan GW Dempfle L. Relative expression software tool (REST) for group-wise comparison and statistical analysis of relative expression results in real-time PCR. Nucleic Acids Res. (2002) 30:e36:36e–336e. doi: 10.1093/nar/30.9.e36, PMID: 11972351 PMC113859

[39] Dabo S Meurs EF. dsRNA-dependent protein kinase PKR and its role in stress, signaling and HCV infection. Viruses. (2012) 4:2598–635. doi: 10.3390/v4112598, PMID: 23202496 PMC3509664

[40] McEwen E Kedersha N Song B Scheuner D Gilks N Han A. Heme-regulated inhibitor kinase-mediated phosphorylation of eukaryotic translation initiation factor 2 inhibits translation, induces stress granule formation, and mediates survival upon arsenite exposure. J Biol Chem. (2005) 280:16925–33. doi: 10.1074/jbc.M412882200, PMID: 15684421

[41] Lu L Han AP Chen JJ. Translation initiation control by heme-regulated eukaryotic initiation factor 2alpha kinase in erythroid cells under cytoplasmic stresses. Mol Cell Biol. (2001) 21:7971–80. doi: 10.1128/MCB.21.23.7971-7980.2001, PMID: 11689689 PMC99965

[42] Walter P Ron D. The unfolded protein response: from stress pathway to homeostatic regulation. Science. (2011) 334:1081–6. doi: 10.1126/science.1209038 22116877

[43] Vattem KM Wek RC. Reinitiation involving upstream ORFs regulates ATF4 mRNA translation in mammalian cells. Proc Natl Acad Sci U S A. (2004) 101:11269–74. doi: 10.1073/pnas.0400541101, PMID: 15277680 PMC509193

[44] Lewerenz J Sato H Albrecht P Henke N Noack R Methner A. Mutation of ATF4 mediates resistance of neuronal cell lines against oxidative stress by inducing xCT expression. Cell Death Differ. (2012) 19:847–58. doi: 10.1038/cdd.2011.165, PMID: 22095285 PMC3321624

[45] Sato H Tamba M Ishii T Bannai S. Cloning and expression of a plasma membrane cystine/glutamate exchange transporter composed of two distinct proteins. J Biol Chem. (1999) 274:11455–8. doi: 10.1074/jbc.274.17.11455, PMID: 10206947

[46] Harding HP Zhang Y Zeng H Novoa I Lu PD Calfon M. An integrated stress response regulates amino acid metabolism and resistance to oxidative stress. Mol Cell. (2003) 11:619–33. doi: 10.1016/S1097-2765(03)00105-9 12667446

[47] Sun Y Pu LY Lu L Wang XH Zhang F Rao JH. N-acetylcysteine attenuates reactive-oxygen-species-mediated endoplasmic reticulum stress during liver ischemia-reperfusion injury. World J Gastroenterol. (2014) 20:15289–98. doi: 10.3748/wjg.v20.i41.15289, PMID: 25386077 PMC4223262

[48] Walters MT Rubin CE Keightley SJ Ward CD Cawley MI. A double-blind, cross-over, study of oral N-acetylcysteine in Sjögren's syndrome. Scand J Rheumatol Suppl. (1986) 61:253–8. 3296153

[49] Zhu H Chen X Chen B Fan J Song W Xie Z. Activating transcription factor 4 mediates a multidrug resistance phenotype of esophageal squamous cell carcinoma cells through transactivation of STAT3 expression. Cancer Lett. (2014) 354:142–52. doi: 10.1016/j.canlet.2014.07.044, PMID: 25130172

[50] Han J Back SH Hur J Lin YH Gildersleeve R Shan J. ER-stress-induced transcriptional regulation increases protein synthesis leading to cell death. Nat Cell Biol. (2013) 15:481–90. doi: 10.1038/ncb2738, PMID: 23624402 PMC3692270

[51] Mimura J Inose-Maruyama A Taniuchi S Kosaka K Yoshida H Yamazaki H. Concomitant Nrf2-and ATF4-activation by Carnosic acid cooperatively induces expression of Cytoprotective genes. Int J Mol Sci. (2019) 20:1706. doi: 10.3390/ijms20071706, PMID: 30959808 PMC6480217

[52] Afonyushkin T Oskolkova OV Philippova M Resink TJ Erne P Binder BR. Oxidized phospholipids regulate expression of ATF4 and VEGF in endothelial cells via NRF2-dependent mechanism: novel point of convergence between electrophilic and unfolded protein stress pathways. Arterioscler Thromb Vasc Biol. (2010) 30:1007–13. doi: 10.1161/ATVBAHA.110.204354, PMID: 20185790

[53] Lam WY Bhattacharya D. Metabolic links between plasma cell survival, secretion, and stress. Trends Immunol. (2018) 39:19–27. doi: 10.1016/j.it.2017.08.007, PMID: 28919256 PMC5748358

[54] Roescher N Tak PP Illei GG. Cytokines in Sjögren's syndrome. Oral Dis. (2009) 15:519–26. doi: 10.1111/j.1601-0825.2009.01582.x, PMID: 19519622 PMC2762015

[55] Houen G Trier NH. Epstein-Barr virus and systemic autoimmune diseases. Front Immunol. (2020) 11:587380. doi: 10.3389/fimmu.2020.587380, PMID: 33488588 PMC7817975

[56] Triantafyllopoulou A Moutsopoulos HM. Autoimmunity and coxsackievirus infection in primary Sjogren's syndrome. Ann N Y Acad Sci. (2005) 1050:389–96. doi: 10.1196/annals.1313.090, PMID: 16014556

[57] Del Papa N Minniti A Lorini M Carbonelli V Maglione W Pignataro F. The role of interferons in the pathogenesis of Sjögren's syndrome and future therapeutic perspectives. Biomol Ther. (2021) 11:251. doi: 10.3390/biom11020251, PMID: 33572487 PMC7916411

[58] Jara D Carvajal P Castro I Barrera MJ Aguilera S González S. Type I interferon dependent hsa-miR-145-5p downregulation modulates MUC1 and TLR4 overexpression in salivary glands from Sjögren's syndrome patients. Front Immunol. (2021) 12:685837. doi: 10.3389/fimmu.2021.685837, PMID: 34149728 PMC8208490

[59] Kim Y Park J Kim S Kim M Kang MG Kwak C. PKR senses nuclear and mitochondrial signals by interacting with endogenous double-stranded RNAs. Mol Cell. (2018) 71:1051–1063.e6. doi: 10.1016/j.molcel.2018.07.029, PMID: 30174290

[60] Barrera MJ Aguilera S Castro I Carvajal P Jara D Molina C. Dysfunctional mitochondria as critical players in the inflammation of autoimmune diseases: potential role in Sjögren's syndrome. Autoimmun Rev. (2021) 20:102867. doi: 10.1016/j.autrev.2021.102867, PMID: 34118452

[61] Lin JH Li H Yasumura D Cohen HR Zhang C Panning B. IRE1 signaling affects cell fate during the unfolded protein response. Science. (2007) 318:944–9. doi: 10.1126/science.1146361, PMID: 17991856 PMC3670588

[62] Cao SS. Epithelial ER stress in Crohn's disease and ulcerative colitis. Inflamm Bowel Dis. (2016) 22:984–93. doi: 10.1097/MIB.0000000000000660, PMID: 26950312

[63] Maida I Zanna P Guida S Ferretta A Cocco T Palese LL. Translational control mechanisms in cutaneous malignant melanoma: the role of eIF2α. J Transl Med. (2019) 17:20. doi: 10.1186/s12967-019-1772-z, PMID: 30634982 PMC6329103

[64] Sato H Nomura S Maebara K Sato K Tamba M Bannai S. Transcriptional control of cystine/glutamate transporter gene by amino acid deprivation. Biochem Biophys Res Commun. (2004) 325:109–16. doi: 10.1016/j.bbrc.2004.10.009, PMID: 15522208

[65] Looms D Tritsaris K Pedersen AM Nauntofte B Dissing S. Nitric oxide signalling in salivary glands. J Oral Pathol Med. (2002) 31:569–84. doi: 10.1034/j.1600-0714.2002.00047.x 12406302

[66] Albano R Raddatz NJ Hjelmhaug J Baker DA Lobner D. Regulation of system xc(−) by pharmacological manipulation of cellular thiols. Oxidative Med Cell Longev. (2015) 2015:269371. doi: 10.1155/2015/269371, PMID: 25949770 PMC4407525

[67] Ye P Mimura J Okada T Sato H Liu T Maruyama A. Nrf2- and ATF4-dependent upregulation of xCT modulates the sensitivity of T24 bladder carcinoma cells to proteasome inhibition. Mol Cell Biol. (2014) 34:3421–34. doi: 10.1128/MCB.00221-14, PMID: 25002527 PMC4135628

[68] Ebrahimiyan H Aslani S Rezaei N Jamshidi A Mahmoudi M. Survivin and autoimmunity; the ins and outs. Immunol Lett. (2018) 193:14–24. doi: 10.1016/j.imlet.2017.11.004 29155234

[69] Palam LR Baird TD Wek RC. Phosphorylation of eIF2 facilitates ribosomal bypass of an inhibitory upstream ORF to enhance CHOP translation. J Biol Chem. (2011) 286:10939–49. doi: 10.1074/jbc.M110.216093, PMID: 21285359 PMC3064149

