# Supplementary material for: The integrated stress response is activated in the salivary glands of Sjögren’s syndrome patients
Source: Front Med (Lausanne). 2023 Mar 23;10:1118703. doi: 10.3389/fmed.2023.1118703 (PMC10079080; doi:10.3389/fmed.2023.1118703)
Supplement: Supplementary file 1 [file Data_Sheet_1.pdf]

# *Supplementary Material*

## The integrated stress response is activated in the salivary glands of Sjögren's syndrome patients

Patricia Carvajal[†], Verónica Bahamondes[†], Daniela Jara[†], Isabel Castro, Soledad Matus, Sergio Aguilera, Claudio Molina, Sergio González, Marcela Hermoso, María-José Barrera[*], María-Julieta González[*]

*** Correspondence:** María-Julieta González: julietagonzal@gmail.com; María-José Barrera: maria.barrera@uss.cl

**1 Supplementary Table 1.** Demographic and serological characteristics of SS-patients and control subjects.

| Parameters | | Control subjects | Patients with primary Sjögren's syndrome |
|---|---|---|---|
| **Numbers of individuals** | | 34 | 41 |
| **Sex, n°. female / n°. male** | | 31/3 | 39/2 |
| **Age, mean (range), years** | | 39.35 (18-68) | 45.49 (21-71) |
| **Focus score [a]** | | | |
| | **1** | 0 | 16 |
| | **2** | 0 | 8 |
| | **≥3** | 0 | 17 |
| **USWSF, mL/15 minutes mean (range)** | | 4.16 (0.2-18.5) | 1.76 (0-5.8) |
| **Ro antibodies n° (%)** | | 0 % | 80.48 % |
| **La antibodies n° (%)** | | 0 % | 48.78 % |
| **Antinuclear antibodies n° (%)** | | 11.7% | 92.68 % |
| **Rheumatoid factor n° (%)** | | 0 % | 48.78 % |
| **ESSDAI mean-median; IQR [25-75]** | | - | 7.88 -6 [4-9.5] |

n°, number; USWSF, Unstimulated whole salivary flow; %, percentage; ESSDAI, EULAR SS disease activity index; EULAR, European League against Rheumatism; IQR, interquartile range.

[a] Number of foci per 4 $mm^2$ of tissue.

2 **Supplementary Table 2.** Sequences of RT-qPCR and MS-HRM primers

| Gene | Accession number | Primer sequences |
|---|---|---|
| **PKR** | NM_002759.3<br>NM_001135651.3<br>NM_001135652.2 | F: 5'-CGACCCTGAGGGTGAATTTCAACT-3'<br>R: 5'-CAGATGCTGGTGCCATGTTTCTTG-3' |
| **PERK** | NM_001313915.2<br>NM_004836.7 | F: 5'-AGGGAATTGGCTCGGGAAAA-3'<br>R: 5'-TGGCCAGTCTGTGCTTTCAT-3' |
| **HRI** | NM_182810.2<br>NM_001134335.1 | F: 5'-GAGTGGGTACTTGTCTGTACGCTT-3'<br>R: 5'-TTCCGGCAACTGACCAGTTCTT-3' |
| **GCN2** | NM_001013703.4 | F: 5'-TCGAGAAGGAAAGGCAGACAGAGA-3'<br>R: 5'-TAGCACACTCACAATGGGAACCAC-3' |
| **eIF2α** | NM_004094.5 | F: 5'-AGCCCTAAGAGCAGGTTTGA-3'<br>R: 5'-ACACACCCCTCTTTTCCTCA-3' |
| **PP1c** | NM_002710.4<br>NM_001244974.2 | F: 5'-AGAGGGTCCAAGCCTGGTAAGAAT-3'<br>R: 5'-TGACTGCTTTCCCCTGTCCACATA-3' |
| **CREP** | NM_032833.5 | F: 5'-GGGAGCCAAGAAAGTGAATGTCCA-3'<br>R: 5'-ACCTGCATCCATCCCTTGCAAA-3' |
| **GADD34** | NM_014330.5 | F: 5'-TTCCGAGTGGCCATCTATGTACCT-3'<br>R: 5'-AAGCGCACCTTTCTGGCCTTTA-3' |
| **ATF4** | NM_001675.4<br>NM_182810.2 | F: 5'-TGGTGAGTGCAAAGAGCTGGAA-3'<br>R: 5'-ACAAGCACATTGACGCTCCTGA-3' |
| **ATF4 met** | S707992* | F: 5'-ATAAAAATTTTTCGTCGGAAAA-3'<br>R: 5'-TAATCAAAATCCGTTCTTTCC-3' |
| **xCT** | NM_014331.4 | F: 5'-TGGGGAGAGTTCTGGTACTGCAAT-3'<br>R: 5'-GAAGCAACTAGAAGCGTGACAGGT-3' |
| **4F2hc** | NM_001012662.3<br>NM_001012664.3<br>NM_001013251.3<br>NM_002394.6 | F: 5'-CCTCATTCTTGGCTGAGTGGCAAA-3'<br>R: 5'-TGCTCCCCAGTAGAACCAGAATCA-3' |
| **Survivin** | NM_194397.3 | F: 5'-CGAACCCCAGACCTGTTTGTATCA-3'<br>R: 5'-GAGTACAGAGGCTGGAGTGCATTT-3' |
| **CHOP** | NM_001195053.1<br>NM_001195054.1<br>NM_001195055.1<br>NM_001195056.1<br>NM_001195057.1<br>NM_004083.6 | F: 5'-AACGGCTCAAGCAGGAAATC-3'<br>R: 5'-TAGCCACTTCTGGGAAAGGT-3' |
| **NRF2** | NM_001145412.3 | F: 5'-ATGCCCTCACCTGCTACTTT-3'<br>R: 5'-TGTTCTGGTGATGCCACACT-3' |
| **h18S** | NM_022551.2 | F: 5’-GATATGCTCATGTGGTGTTG-3’<br>R: 5’-AATCTTCTTCAGTCGCTCCA-3’ |

*Accession number http://switchgeargenomics.com/

**Supplementary Table 3.** Antibodies used for Western blot and immunofluorescence

| Antibodies | Source | Dilution | Company |
|---|---|---|---|
| Anti-PKR, polyclonal, 3072, IgG. | Rabbit | WB: 1:1.000<br>IF: 1:200 | Cell Signaling technology®, Inc. |
| Anti-pPKR (T446), monoclonal, ab32036, IgG | Rabbit | WB: 1:1.000<br>IF: 1:200 | Abcam plc. |
| Anti-PERK, monoclonal, C33E10, IgG. | Rabbit | WB 1:1.000<br>IF: 1:75 | Cell Signaling technology®, Inc. |
| Anti-pPERK(Thr981), polyclonal.sc-32577. | Rabbit | WB 1:1.000<br>IF: 1:200 | Santa Cruz Biotechnology, Inc. |
| Anti-HRI, polyclonal, ab28530, IgG | Rabbit | WB: 1:1.000 | Abcam plc. |
| Anti-GCN2, polyclonal, 3302. | Rabbit | WB: 1:1.000 | Cell Signaling technology®, Inc. |
| Anti-pGCN2 (T899), monoclonal, ab75836 IgG | Rabbit | WB: 1:1.000 | Abcam plc. |
| Anti-eIF2α, polyclonal, 9722 | Rabbit | WB 1:1.000<br>IF: 1:75 | Cell Signaling technology®, Inc. |
| Anti-peIF2a (Ser51), monoclonal, 3398 | Rabbit | WB 1:1.000<br>IF 1:100 | Cell Signaling technology®, Inc. |
| Anti-PPP1CC, polyclonal, NBP1-32858, IgG | Rabbit | WB: 1:1.000 | Novus Biologicals, EEUU. |
| Anti-CReP, polyclonal, 14634-1-AP, IgG | Rabbit | WB: 1:1.000 | Proteintech Group, Inc. |
| Anti-Gadd34, polyclonal, NB100-778, IgG | Goat | WB: 1:1.000 | Novus Biologicals, EEUU. |
| Anti-ATF4, monoclonal, D4B8, IgG. | Rabbit | WB 1:1.000 | Cell Signaling technology®, Inc. |
| Anti-CREB2 (ATF4) (C20), c-200. Polyclonal. | Rabbit | IF 1:100 | Santa Cruz Biotechnology, Inc. |
| Anti-xCT, NB300-318, polyclonal. | Rabbit | WB 1:1.000<br>IF: 1:200 | Novus Biologicals, EEUU. |
| Anti-human survivin, polyclonal. | Rabbit | WB 1:1.000 | R&D Systems, Inc. |
| Anti-CHOP, monoclonal, L63F7, IgG2a. | Mouse | WB 1:1.000<br>IF: 1:100 | Cell Signaling technology®, Inc. |
| Anti-NRF2, polyclonal, bs-1074R, IgG | Rabbit | WB: 1:1.000 | Bioss |
| Anti-NRF2 (D1C9), monoclonal, IgG. | Rabbit | IF: 1:100 | Cell Signaling technology®, Inc. |
| Anti-pNRF2 (Ser40), polyclonal, bs-2013R, IgG | Rabbit | WB: 1:1.000 | Bioss |
| Anti-KEAP1,monoclonal, 8047S | Rabbit | WB: 1:1.000 | Cell Signaling technology®, Inc. |
| Anti-β-actin, monoclonal (BA3R) | Mouse | WB 1:20.000 | Invitrogen |

4 **Supplementary Table 4.** Spearman's rank correlation coefficients between clinical parameters and molecular determinations of SS-patients and control subjects.

| Parameters | R | p |
|---|---|---|
| USWSF - p-PERK/PERK ratio | -0.466 | 0.017 |
| USWSF - PP1c protein | 0.648 | < 0.001 |
| USWSF - CREP protein | 0.481 | 0.023 |
| USWSF - xCT protein | -0.788 | 0.001 |
| Ro antibodies - p-PERK/PERK ratio | 0.569 | < 0.001 |
| Ro antibodies - PKR mRNA | 0.590 | 0.003 |
| Ro antibodies - PP1c protein | -0.682 | < 0.001 |
| Ro antibodies - ATF4 protein | 0.751 | < 0.001 |
| Ro antibodies - xCT protein | 0.500 | 0.021 |
| La antibodies - p-PERK/PERK ratio | 0.431 | 0.014 |
| La antibodies - PP1c protein | -0.524 | 0.010 |
| La antibodies - ATF4 protein | 0.561 | < 0.001 |
| La antibodies - xCT protein | 0.557 | 0.009 |
| Rheumatoid Factor - p-PERK/PERK ratio | 0.395 | 0.025 |
| Rheumatoid Factor - CREP protein | -0.504 | 0.017 |
| Rheumatoid Factor - ATF4 protein | 0.436 | 0.011 |
| Antinuclear antibodies - p-PERK/PERK ratio | 0.624 | < 0.001 |
| Antinuclear antibodies - PP1c mRNA | -0.482 | 0.020 |
| Antinuclear antibodies - PP1c protein | -0.564 | < 0.001 |
| Antinuclear antibodies - ATF4 protein | 0.684 | < 0.001 |
| Focus score - p-PERK/PERK ratio | 0.583 | < 0.001 |
| Focus score - PKR mRNA | 0.496 | 0.016 |
| Focus score - PP1c mRNA | -0.565 | 0.005 |
| Focus score - PP1c protein | -0.755 | < 0.001 |
| Focus score - CREP protein | -0.444 | 0.039 |
| Focus score - ATF4 protein | 0.690 | <0.001 |
| Focus score - xCT protein | 0.475 | 0.030 |
| ESSDAI - p-PERK/PERK ratio | 0.482 | 0.023 |
| ESSDAI - PP1c protein | -0.609 | 0.003 |
| ESSDAI - ATF4 protein | 0.661 | <0.001 |
| p-PERK/PERK ratio - PP1c protein | -0.539 | 0.041 |
| PKR protein - eIF2α protein | 0.676 | 0.003 |
| PKR protein - p-eIF2α protein | 0.538 | 0.023 |
| p-PKR protein - p-eIF2α protein | 0.616 | 0.008 |
| p-PKR protein – PERK protein | 0.745 | 0.003 |
| PP1c protein - ATF4 protein | -0.621 | 0.016 |
| ATF4 mRNA – ATF4 promoter methylation | -0.566 | 0.022 |

*p<0.05. USWSF: unstimulated whole salivary flow.

## 5 Supplementary Figures

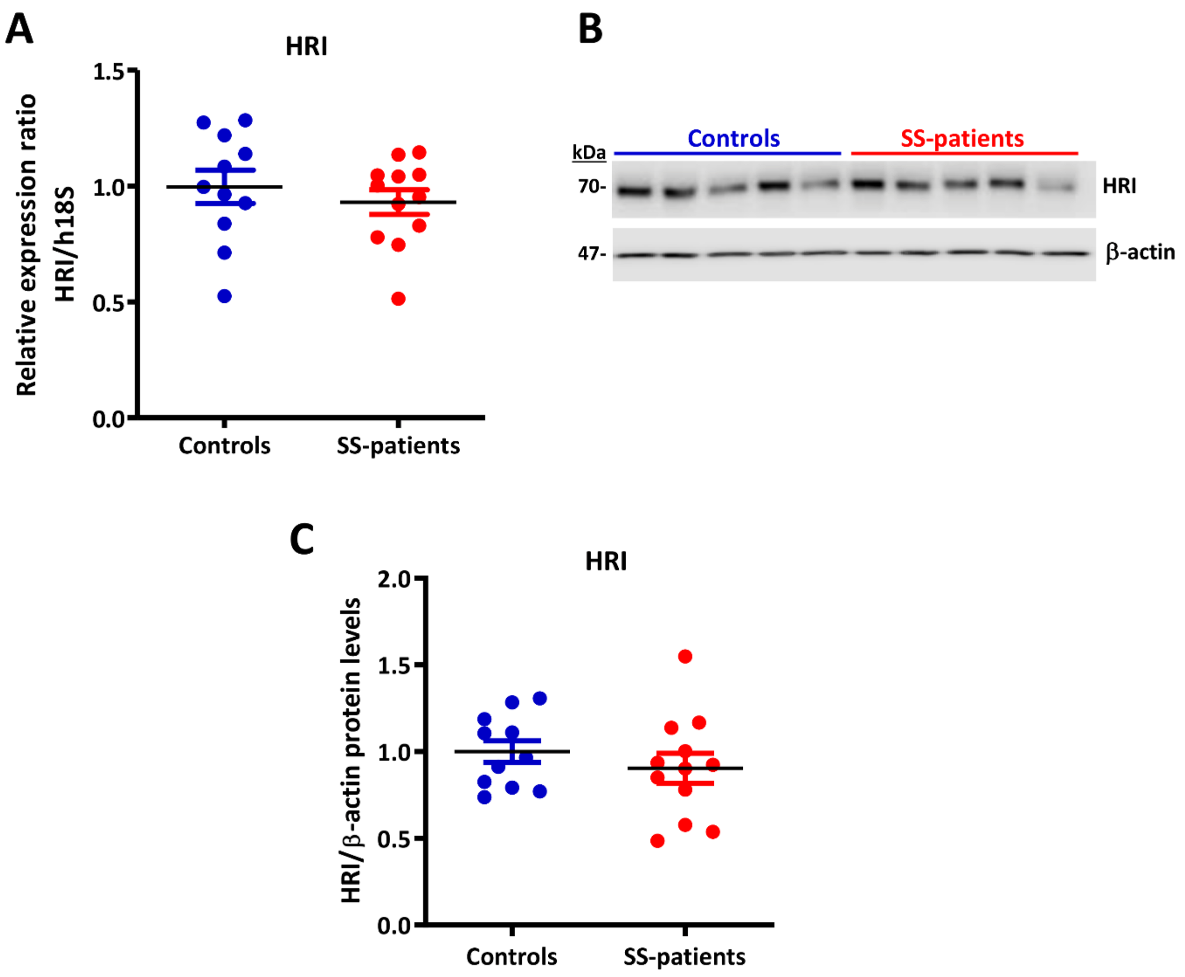


**Supplementary Figure 1. Expression of HRI in LSGs from control and SS-patients. A,** dot plot showing HRI transcript levels relative to h18S in controls (C) and SS-patients (P) (n= 11C, 12P). **B,** representative images of HRI immunoblots from control and SS-patients using β-actin as a loading control. **C,** dot plot showing densitometric analysis of HRI (n= 11C, 12C). These experiments were repeated at least three times (*). P values lower than 0.05 were considered significant.

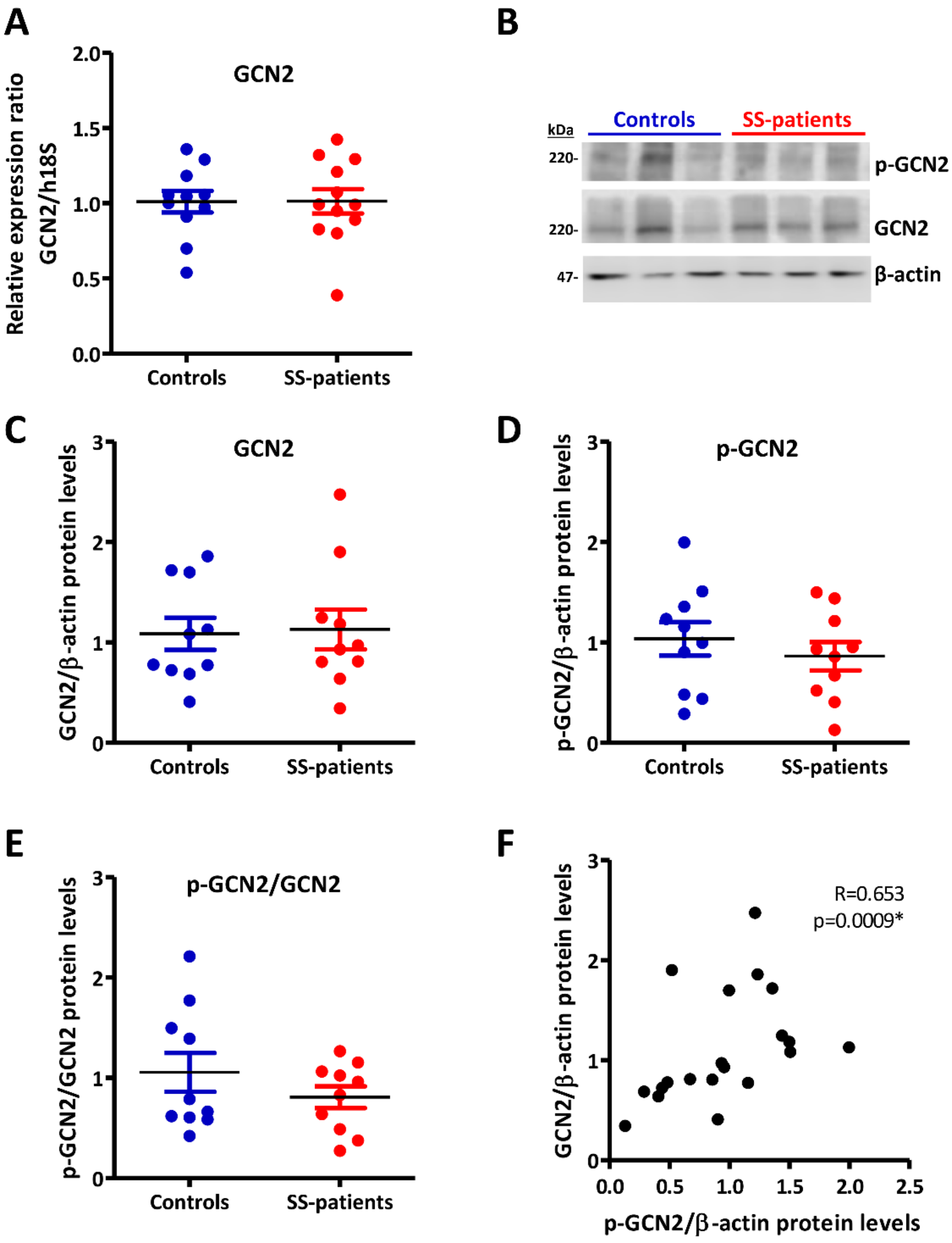


**Supplementary Figure 2. Expression and activation of GCN2 in LSGs from control and SS-patients. A,** dot plot showing GCN2 transcript levels relative to h18S in controls (C) and SS-patients (P) (n= 11C, 12P). **B,** representative images of p-GCN2 and GCN2 immunoblots from control and SS-patients using β-actin as a loading control. **C,** dot plot showing densitometric analysis of GCN2 (n= 10C, 10P). **D,** dot plot showing densitometric analysis of p-GCN2 (n=10C, 10P). **E,** dot plot showing the p-GCN2/GCN2 ratio (n= 10C, 10P). **F,** Spearman's correlation between p-GCN2 and GCN2 protein levels. These experiments were repeated at least three times (*). P values lower than 0.05 were considered significant.

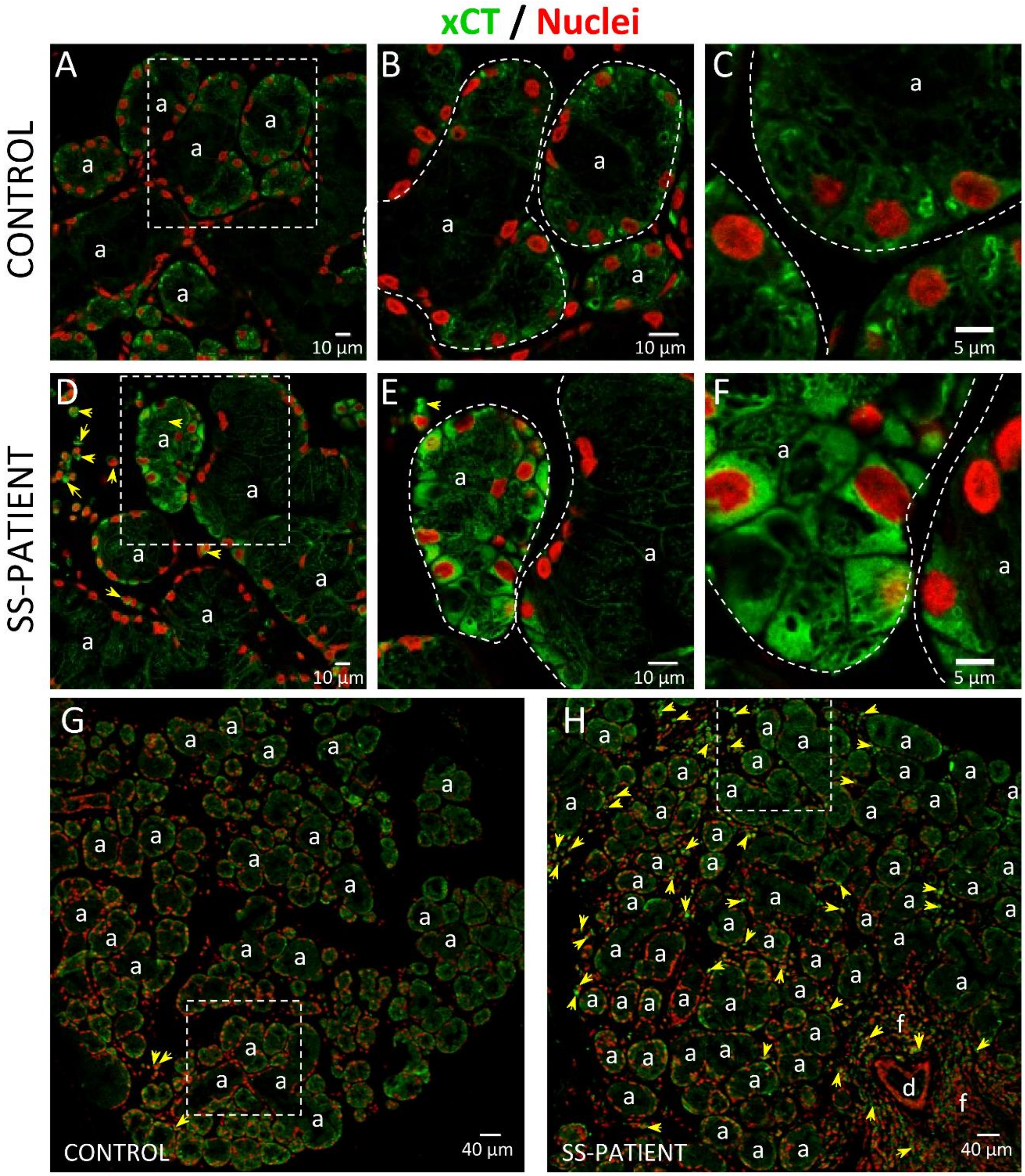


**Supplementary Figure 3. Localization of xCT in LSGs from control and SS-patients. A-C, G,** xCT (green) staining was mainly observed in the cytoplasm of epithelial cells in LSGs from control subjects. **D-F, H**, stronger xCT (green) staining was observed in the cytoplasm of epithelial and plasma cells (yellow arrows) in LSGs from SS-patients. Differences in xCT staining intensity were observed between neighbour acinar cells. **A** and **D** higher magnifications of regions surrounded by broken lines in G and H, respectively. **B-C** and **E-F** higher magnifications of regions surrounded by broken lines in A and B, respectively. Nuclei (red) were counterstained with Hoechst-33342. a: acini; d: duct; f: focus of inflammatory cells. Bars A, B, D and E: 10 µm; C and F: 5 µm; G and H: 40 µm.

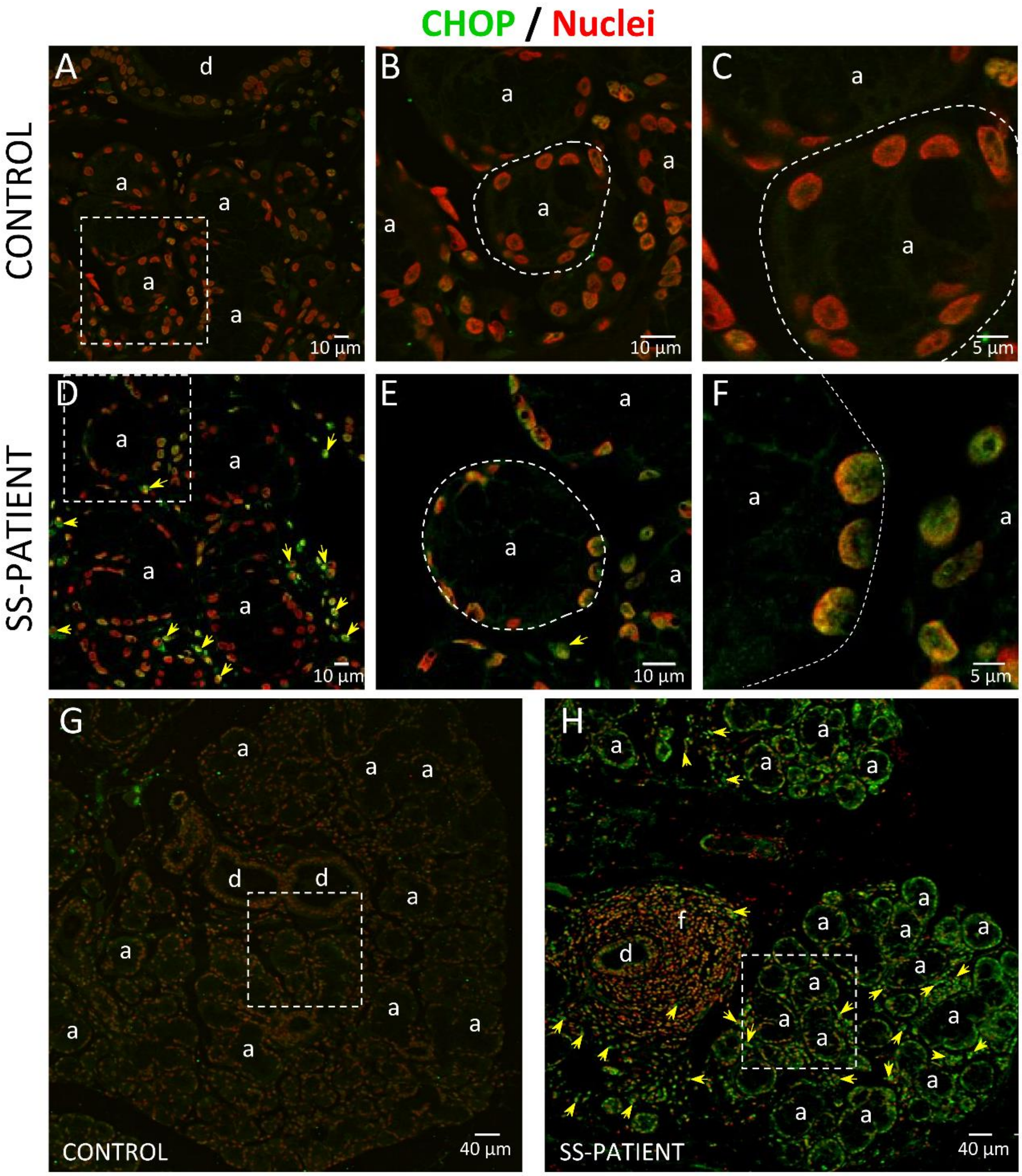


**Supplementary Figure 4. Localization of CHOP in LSGs from control and SS-patients. A-C, G,** CHOP (green) staining was almost undetectable in epithelial cells in LSG from control subjects. **D-F, H**, stronger CHOP (green) staining was observed in the nuclei and cytoplasm of epithelial and inflammatory cells (yellow arrows) in LSGs from SS-patients. **A** and **D** higher magnifications of regions surrounded by broken lines in G and H, respectively. **B-C** and **E-F** higher magnifications of regions surrounded by broken lines in A and B, respectively. Nuclei (red) were counterstained with Hoechst-33342. a: acini; d: duct; f: focus of inflammatory cells. Bars A, B, D and E: 10 µm; C and F: 5 µm; G and H: 40 µm.
